# Curing spurious magneto‐mechanical coupling in soft non‐magnetic materials

**DOI:** 10.1002/nme.7210

**Published:** 2023-02-12

**Authors:** Matthias Rambausek, Joachim Schöberl

**Affiliations:** ^1^ Institute of Analysis and Scientific Computing TU Wien Vienna Austria

**Keywords:** finite elasticity, finite magneto‐elasticity, magnetostatics, numerical artifact, spurious coupling

## Abstract

The present work is concerned with the issue of spurious coupling effects that are pervasive in fully coupled magneto‐mechanical finite element simulations involving very soft non‐magnetic or air‐like media. We first address the characterization of the spurious magneto‐mechanical effects and their intuitive interpretation based on energy considerations. Then, as main contribution, we propose two new cures for the issue under consideration that completely prune the undesired spurious magneto‐mechanical coupling in non‐magnetic media. The proposed methods are compared with established methods in the context of magnetic bodies embedded in (i) air or vacuum and (ii) very soft elastic non‐magnetic media. The comparison shows that the proposed approaches are accurate and effective. They, furthermore, allow for a consistent linearization of the coupled boundary value problems, which is crucial for the simulation of compliant structures. For reproducibility and accessibility of the proposed methods, we provide our implementations with Netgen/NGSolve as well as all codes necessary for the reproduction of our results as supplementary material.

## 1 INTRODUCTION

In this work we address the fundamental numerical issue of spurious magnetic coupling in full‐field simulations of extremely compliant magnetic bodies and structures. The bodies that we have in mind are composites consisting of an elastomer or (hydro)gel matrix with almost rigid ferromagnetic inclusions, that is, magnetorheological elastomers (MREs) or magneto‐active gels (MAGs). For experimental data on MREs we refer to Jolly et al.,^1^ Bednarek,^2^ Ginder et al.,^3^ Martin et al.,^4^ Böse and Röder,^5^ Danas et al.,^6^ Bodelot and Danas^7^ and Garcia‐Gonzalez et al.^8^ as well as to the extensive review by Bastola and Hossain.^9^ These findings have inspired many interesting applications of MREs such magnetically actuated valves,^10^ magnetically tunable dampers^11^ and vibration isolators^12^, ^13^, ^14^ as well as micropumps.^15^ With matrix materials becoming softer and bodies and structures becoming more compliant, new opportunities for applications open up in medical and biological contexts.^16^, ^17^ However, in this subclass of MREs well‐established experimental, numerical and theoretical approaches face new challenges. For pioneering experimental data we refer to Nikitin et al.,^18^ Stepanov et al.^19^, ^20^ and rather recently Moreno‐Mateos et al.,^21^, ^22^ which set several opportunities for future research. A first attempt to connect simulation and experiments in this particular domain has been made by Lucarini et al.^23^ MAGs have been pioneered by Zrínyi et al.^24^ Measurements and experimental data shows that MAGs exhibit changes of stiffness when exposed to magnetic fields^24^, ^25^, ^26^, ^27^ and may serve as bio‐compatible soft actuators.^28^, ^29^ They have been proposed for numerous novel biomedical applications,^30^ for example drug delivery^31^, ^32^ and modulation of cell activity.^33^ In context of the materials under consideration, full‐field simulations context are paramount for the understanding and the prediction of the strongly coupled nonlinear magneto‐mechanical response of the materials and bodies of interest. The theoretical groundwork has been established in the second half of the 20th century^34^, ^35^, ^36^ and modernized roughly two decades ago.^37^, ^38^, ^39^, ^40^, ^41^, ^42^ With the availability of corresponding finite element methods^*^, for example, References 43, 44, 45 numerical simulations have been widely applied to augment purely theoretical as well as experimental data and insight. In this regard it is important to account for the composite nature of magnetorheological elastomers with computational means^46^, ^47^, ^48^, ^49^, ^50^, ^51^, ^52^, ^53^ or (semi‐) analytical approaches.^54^, ^55^, ^56^, ^57^, ^58^, ^59^, ^60^, ^61^ For explicit models with the capability to fit computational homogenization results we mention Mukherjee et al.^62^ and Kalina et al.^48^ Models fitted against experimental data have been reported by Danas et al.^6^ and Haldar,^63^ whereby the latter takes viscoelastic effects into account.

A particular breed of MREs based on remanently magnetized particles dates back at least to the exploratory experiments of Stepanov et al.^64^ Such “hard” MREs (h‐MREs) have recently received increased attention thanks to advances in additive manufacturing of h‐MRE *structures*.^65^ Promising fields of application of this new technology are soft robotics^66^, ^67^, ^68^ and self‐healing elastomers.^69^ There exist various works on numerical modeling of h‐MREs under assumption of a *prescribed* remanent magnetization.^70^, ^71^, ^72^, ^73^, ^74^ The resulting models reduce to finite elasticity with particular loading conditions. In contrast to that, the important aspect of ferromagnetic evolution in the particles, for example, during the magnetization process of h‐MREs, has been accounted only in a few methodological contributions.^75^, ^76^ Macroscopic material models that are able to reproduce microstructural simulations of h‐MREs have been developed recently.^77^, ^78^ For comprehensive reviews on h‐MREs we refer to Lucarini et al.^79^ but also to Wu et al.^80^ where both (magnetically) “soft” MREs and h‐MREs were considered. Alongside these developments in the context of MREs, multiphysics simulation frameworks and models for MAGs^81^, ^82^ have been established in recent years.

**FIGURE 1:**
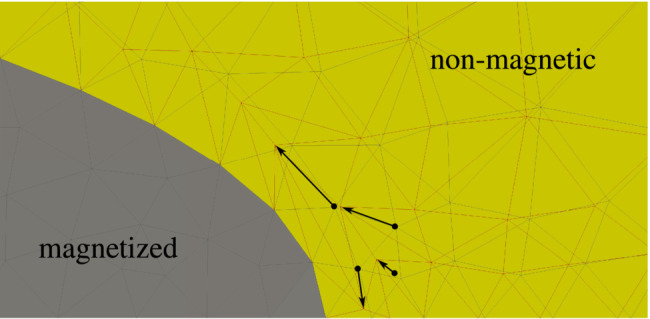
A spatially fixed, rigid and magnetized body (gray) embedded in a soft non‐magnetic material (yellow). The shear and bulk moduli employed for the non‐magnetic domain are μ=7.142kPa and κ=33.333kPa, respectively. The magnetized body cannot deform due to its rigidity. The non‐magnetic domain, however, deforms in response to magnetic fields (red vs. gray mesh). This is physically impossible and, thus, must be a spurious effect from numerics to which we refer to as *spurious deformation*. The figure is taken from Chapter. 9.3.1 (Figure 9.17a) of the author's doctoral thesis.^83^

No matter whether “soft” or “hard” MREs or MAGs are considered in simulations, one almost always encounters the pervasive issue that will be addressed by this contribution: spurious magneto‐mechanical coupling in sufficiently soft or compliant magnetoactive bodies as depicted in Figure 1.^83^ There one can observe excessive deformation in the non‐magnetic material (yellow) embedding a clamped, *rigid* magnetized body (gray) where no deformation at all is expected according to the laws of physics. These effects are ubiquitous but only become relevant in extremely soft materials. This can be for example the case when a sufficiently soft non‐magnetic medium is employed to model the air surrounding some magnetic specimen.^50^ However, it may also be observed when increasingly soft^†^ matrix materials are employed as is the case for example in the experiments of Reference 21. While both situations have the commonality of an extremely soft non‐magnetic material being present in the boundary value problem (BVP), in the first case this is purely for convenient modeling of a medium with no significant stiffness at all, in the second case the soft material is physical reality. Due to that, in the former case, one has more modeling and methodological freedom than in the latter. In fact, Pelteret et al.^84^ proposed an elegant staggered approach to account for air or vacuum surrounding a magnetic body that does not make use of an auxiliary soft material and hence does not suffer from spurious coupling in the present sense. However, their approach is not applicable when a soft elastic material is indeed physically present. Also, due to its partially decoupled nature and the inherent inconsistent linearization of the discretized BVP, it suffers from convergence issues in the case of highly compliant structures. As a second method that successfully circumvents spurious coupling effects we mention the staggered approach of Liu et al.^85^ Their approach is based on surface currents computed in a purely magnetic BVP. In a second step, these currents are employed to compute magneto‐mechanical tractions on boundaries of magnetic bodies for the mechanical subproblem. Due to the decoupled magnetostatic and mechanical subproblems, the scheme requires a certain number of iterations between both of them to account for the strong coupling and attain convergence. While the procedure is effective for the analysis of slender magneto‐active structures, its staggered nature and the explicit use of electric currents for force computation render the scheme less suited for adoption in existing fully coupled (monolithic) methods. A third approach for the modeling of surrounding air in finite element simulation are non‐local constraints that bind the motion in the air domain to the motion of the boundary of the magnetic bodies under consideration.^77^, ^86^ While this method does not introduce artificial stiffness and linearizes properly, it leads to a much denser linear system. Furthermore, it is difficult to implement for general geometries and arrangements of multiple magnetic bodies. For some additional insights on existing monolithic and staggered schemes we refer to Rambausek et al.^76^

The methods proposed in this contribution exploit the non‐magnetic nature of the soft material (surrounding air or soft carrier/matrix material) to obtain formulations that integrate well with existing magneto‐elasticity frameworks. That is, it is not much more difficult to implement than the naive monolithic schemes, for both the surrounding air and the soft matrix case. Furthermore, the schemes obtained linearize properly and at the same time do not suffer from spurious coupling and are thus promising candidates for widespread adoption. To facilitate this process we provide our implementation with Netgen/NGSolve^87^, ^88^, ^89^ as well as codes for our numerical examples as Supplementary Material.

The remainder of this contribution is structured as follows. In Section 2, we outline the fundamental equations of finite magneto‐elasticity as well as important considerations on magneto‐mechanical interactions. Section 3 then presents the spurious magneto‐mechanical coupling that is the motivation for the present work and discusses the underlying general pattern. Then, in Section 4 we arrive at the heart of our contribution where we propose two approaches that eliminate spurious magneto‐mechanical interactions in non‐magnetic domains. We sketch numerical implementations of the schemes and assess their accuracy by a comparison with existing methods as well as their convergence under mesh refinement. After that we showcase both proposed approaches in a challenging example involving a magnetic solid and a very soft non‐magnetic solid under gravitational and magnetic loads embedded in an air‐like medium. We close our contribution with the conclusion in Section 5.

**FIGURE 2:**
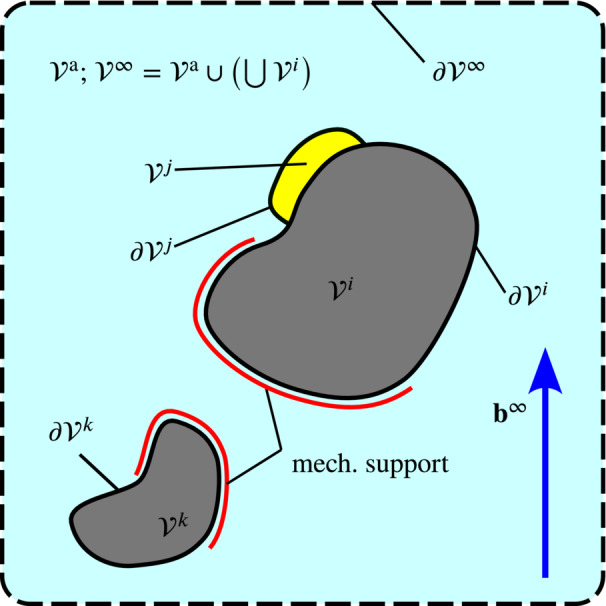
The overall setting considered in this work. Magnetic bodies (gray; ${ℬ}^{i,k}$) and non‐magnetic bodies (yellow; ${ℬ}^{j}$) in “free” space (light cyan; vacuum, air‐like medium) exposed to a uniform external magnetic field $b^{\infty }$. We employ the symbol ${ℬ}^{\infty }$ for the full domain including all sub‐bodies and ${ℬ}^{a}$ for the “remaining” empty or air‐like domain.

## 2 THEORETICAL BACKGROUND

For the problem of spurious magneto‐mechanical coupling investigated in this work, we restrict ourselves to quasi‐static finite elasticity coupled to magnetostatics in absence of free currents. We first recall the fundamental theory for the mechanical part, then the magnetostatic and finally bring both fields together. Figure 2 provides an overview of the setting considered. There we depict magnetic bodies (gray; ${ℬ}^{i,k}$) and non‐magnetic bodies (yellow; ${ℬ}^{j}$) embedded in “empty” space ${ℬ}^{a}$ (light cyan; vacuum, air‐like medium). The bodies are exposed to a uniform external magnetic field $b^{\infty }$ and may have mechanical support. While not indicated, we may of course consider mechanical body forces like gravity as well. The “full” domain consisting of ${ℬ}^{\infty }$ and all (solid) bodies ${ℬ}^{i}$ is denoted as ${ℬ}^{\infty }$. The (computational) outer boundary $\partial {ℬ}^{\infty }$ is indicated by a dashed line. Physically, there is no boundary but in finite element simulations the infinite space domain is truncated^‡^ at a larger distance from the bodies under consideration.^45^, ^50^, ^92^, ^93^ For definiteness, we mechanically fix the outer boundary $\partial {ℬ}^{\infty }$.

### 2.1 Quasi‐static finite hyperelasticity

In the context of finite strains we have to distinguish between the *Eulerian* or *current configuration* and the *Lagrangian* or *reference* ^§^ *configuration*. The former simply refers to the (deformation) state of media as an external observed perceives them, whereas the latter takes the perspective of material points. Following a common convention, we denote quantities in the Eulerian configuration by lowercase symbols, whereas uppercase symbols are employed for the Lagrangian configuration. In some instances, we employ subscript “0” for Lagrangian and subscript “t” for Eulerian quantities for definiteness. The Eulerian and the Lagrangian configuration are connected through the map φ, that assigns to each Lagrangian position X (a material point) a Eulerian position x=φ(X). The corresponding tangent map or Jacobian $F=\frac{\partial \varphi }{\partial X}$. In Cartesian coordinates F is often written as

(1) F(X)=Gradφ(X)⇒CurlF≡0,

and commonly referred to as deformation gradient. Its determinant J=DetF is a measure for the change of volume elements

(2) $$\begin{array}{ll} \;dv=J\;dV, & \end{array}$$

where dv is the Eulerian and dV the Lagrangian volume element.

On the kinetic side, the balance of momentum in a quasi‐static setting reads

(3) $$\text{div}\;\sigma +f^{\;b}=0\;\text{in}\;{ℬ}_{t}\;\;\Leftrightarrow \;\;\text{Div}\;P+f_{0}^{\;b}=0\;\text{in}\;{ℬ}_{0},$$

in the Eulerian and Lagrangian setting, respectively, where σ denotes the Cauchy stress, P the “first Piola‐Kirchhoff stress” and $f^{\;b}$ denotes the body force density per Eulerian volume. The surface‐integral‐preserving Piola‐type transform relating σ and P is

(4) $$\sigma (X)=J^{-1}P\cdot F^{T}.$$

The Eulerian body force density $f^{\;b}$ transforms as

(5) $$f^{\;b}(X)=J^{-1}f_{0}^{\;b},$$

The corresponding boundary conditions are given as

(6) $$\sigma \cdot n=t\;\text{on}\;\partial {ℬ}_{t}\;\Leftrightarrow \;P\cdot N=\left(J\;\sqrt{\left(F^{T}\cdot N)\cdot (F^{T}\cdot N\right)}\right)t\;\text{on}\;\partial {ℬ}_{0},$$

where t is a given Eulerian traction, that is, a surface force density per Eulerian area and n is the outward‐pointing unit normal. The complicated factor on the right is the ratio of area elements da/dA which transforms the Eulerian to a Lagrangian traction.

In the context of hyperelasticity, the stresses are obtained as partial derivatives of a strain energy density per mass ψ(F)

(7) $$\sigma =\rho \frac{\partial \psi }{\partial F}\;\text{and}\;P=\rho_{0}\frac{\partial \psi }{\partial F}\cdot F^{T},$$

where ρ and $\rho_{0}$ are the mass densities per current (Eulerian) and initial (Lagrangian) volume. The latter are related through

(8) $$\begin{array}{ll} \rho =J^{-1}\rho_{0}. & \end{array}$$

As the final modeling ingredient we introduce the strain energy density per Lagrangian volume $\Psi_{0}(F)=\rho_{0}\psi (F)$, where $\rho_{0}$ is the respective mass density. Correspondingly, the strain energy density per Eulerian volume is $\Psi_{t}(F)=\rho \psi (F)$.

The balance of momentum is accompanied the balance of moment of momentum expressed as

(9) $$\sigma^{T}=\sigma ,$$

that is, the symmetry of the *Cauchy* stress. In the present setting, (9) is ensured by an objective (material frame indifferent) strain energy density. Material frame indifference is achieve, for example, by parameterizing the strain energy density in terms of the “right Cauchy‐Green” tensor

(10) $$C=F^{T}\cdot F,$$

such that $\Psi {\left(F\right)}_{0}=\Psi_{0}(C(F))$ which yields

(11) $$\begin{array}{ll} \sigma =J^{-1}\frac{\partial \Psi_{0}}{\partial F}\cdot F^{T}=2\;J^{-1}F\cdot \frac{\partial \Psi_{0}}{\partial C}\cdot F^{T}. & \end{array}$$

The last term clearly is symmetric and thus σ is symmetric too.

Considering the case where the external volume and surface force densities are *conservative*, we introduce the respective loading potential densities (per Eulerian volume) $l^{f}$ and $l^{t}$ via

(12) $$f^{\;b}=-\frac{\partial l^{f}}{\partial \varphi }\;\text{and}\;t=-\frac{\partial l^{t}}{\partial \varphi }.$$

With these at hand, we may formulate a variational principle for finite hyperelasticity

(13) $$\begin{array}{ll} \hat{\varphi }=\arg\underset{\varphi }{\inf}\left\{\int_{{ℬ}_{t}}\rho \psi (C)\;dv+\int_{{ℬ}_{t}}l^{f}\;dv+\int_{\partial {ℬ}_{t}}l^{t}\;da\right\}, & \end{array}$$

where we remark that the integrals can be individually transformed to the Lagrangian configuration for computational convenience with the help of (2), (8) and the area transform factor in (6). For example, the fully transformed version reads

(14) $$\begin{array}{ll} \hat{\varphi }=\arg\underset{\varphi }{\inf}\left\{\int_{{ℬ}_{0}}\rho_{0}\psi (C)\;dV+\int_{{ℬ}_{0}}l_{0}^{f}\;dV+\int_{\partial {ℬ}_{0}}l_{0}^{t}\;dA\right\}. & \end{array}$$

### 2.2 Magnetostatics

The magnetostatic field equations reduced to the scope of the present work read

(15a) $$\text{div}\;b=0\;\text{in}\;{ℬ}_{t}^{\infty }\;b\cdot n=b^{\infty }\cdot n\;\text{on}\;\partial {ℬ}_{t}^{\infty },$$

and

(15b) $$\text{curl}\;h=0\;\text{in}\;{ℬ}_{t}^{\infty }\;h\times n=h^{\infty }\times n\;\text{on}\;\partial {ℬ}_{t}^{\infty },$$

where b is the magnetic b‐field (magnetic flux), h is magnetic h‐field (magnetic intensity). From (15) it is clear that b and h can be expressed in terms of potentials as

(16) b=curlaandh=−gradϕ,

with a being the (magnetic) vector potential and ϕ the auxiliary *scalar* magnetic potential. While not of real physical significance, ϕ is particularly convenient for computations.

It is possible to show that the equations introduced so far can be obtained from two equivalent variational principles. The first, to which we refer as magnetic energy principle, reads

(17) $$\begin{array}{ll} \hat{a}=\arg\underset{a}{\inf}\left\{\int_{{ℬ}^{\infty }}\Psi_{t}^{b}(x,b=\text{curl}\;a)\;dv-\int_{\partial {ℬ}_{t}^{\infty }}(h^{\infty }\times n)\cdot a\;da\right\}, & \end{array}$$

where $\Psi_{t}^{b}(x,b)$ is the magnetic energy density per Eulerian volume, a quantity to be specified depending on the medium occupying the respective point x in space. At a solution $\hat{a}$ we have

(18) $$\text{curl}\;h=0\;\text{with}\;h=\frac{\partial \Psi_{t}^{b}}{\partial b}.$$

In order to end up with a *fully determined* ^¶^ problem for $\hat{a}$, *in three dimensions* one not only needs to specify essential boundary conditions on a but also a *gauge* condition, for example, the Coulomb gauge diva=0, as an additional constraint.^43^, ^94^

Dual to (17) we have the magnetic *co*‐energy principle

(19) $$\begin{array}{ll} \hat{\phi }=\arg\underset{\phi }{\sup}\left\{\int_{{ℬ}_{t}^{\infty }}\Psi_{t}^{h}(x,h=-\text{grad}\;\phi )\;dv-\int_{\partial {ℬ}_{t}^{\infty }}(b^{\infty }\cdot n)\;\phi \;da\right\}, & \end{array}$$

where ψ(h) is the magnetic co‐energy density, which is obtained from ψ(b) through a Legendre‐Fenchel transform

(20) $$\begin{array}{ll} \Psi_{t}^{h}(h)=\underset{b}{\inf}\left\{-b\cdot h+\Psi_{t}^{b}(b)\right\}. & \end{array}$$

At a solution $\hat{\phi }$ we have

(21) $$\text{div}\;b=0\;\text{with}\;b=-\frac{\partial \Psi_{t}^{h}}{\partial h}.$$

We remark that essential boundary conditions on ϕ are sufficient to uniquely determine ϕ in terms of h and (16)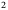. Thus, no gauge fixing is needed in the context of (19).

### 2.3 Coupled magnetoelasticity

In order to couple magnetostatics to finite elasticity we establish the following transforms

(22a) $$\begin{array}{ll} & \phi (X)=\phi (x)\circ \varphi (X), \end{array}$$

(22b) $$\begin{array}{llllll} & h(X)=-\text{grad}\;(\phi (\varphi (X)))=F^{T}\cdot H(X)\; & \text{with} & \; & H=-\text{Grad}\;(\phi (X)), & \end{array}$$

(22c) $$\begin{array}{ll} & \Psi_{t}^{h}(h(X),X)=J^{-1}\Psi_{0}^{h}(h=F^{T}\cdot H,X), \end{array}$$

(22d) $$\begin{array}{ll} & a(X)=F^{T}\cdot A(X), \end{array}$$

(22e) $$\begin{array}{llllll} & b(X)=\text{curl}\;(a(X))=J^{-1}F\cdot B(X)\; & \text{with} & \; & B=\text{Curl}\;(A(X)), & \end{array}$$

(22f) $$\begin{array}{ll} & \Psi_{t}^{b}(b(X),X)=J^{-1}\Psi_{0}^{b}(b=J^{-1}F\cdot B,X). \end{array}$$

We note that the transforms involving $F^{-T}$ preserve line integrals, those involving $J^{-1}F$ preserve surface integrals and those involving $J^{-1}$ preserve volume integrals.

In what follows, we exploit that any material‐frame indifferent $\Psi_{0}^{h}(h=F^{T}\cdot H,X)$ can be recast to the form $\Psi_{0}^{H}(C,H,X)$ and $\Psi_{0}^{b}(b=J^{-1}F\cdot B,X)$ to $\Psi_{0}^{B}(C,B,X)$.^77^, ^78^ The parameterizations of $\Psi_{0}^{H}$ and $\Psi_{0}^{B}$ have the advantage that they directly generalize to the magneto‐mechanical case since they depend on both (mechanical) kinematic and magnetic quantities. With these at hand, we combine the mechanical (14) and magnetic principles (17) and (19) to the coupled variational principles^95^

(23) $$\begin{array}{ll} \left\{\hat{\varphi },\hat{A}\right\}=\arg\underset{\varphi ,A}{\inf}\left\{\int_{{ℬ}_{0}^{\infty }}\Psi_{0}^{B}(C,B)\;dV-\int_{\partial {ℬ}_{0}^{\infty }}(h^{\infty }\times N)\cdot A\;dA+\int_{{ℬ}_{0}}l_{0}^{f}\;dV+\int_{\partial {ℬ}_{0}}l_{0}^{t}\;dV\right\}, & \end{array}$$

and

(24) $$\begin{array}{ll} \left\{\hat{\varphi },\hat{\phi }\right\}=\arg\underset{\varphi }{\inf}\underset{\phi }{\sup}\left\{\int_{{ℬ}_{0}^{\infty }}\Psi_{0}^{H}(C,H)\;dV-\int_{\partial {ℬ}_{0}^{\infty }}(b^{\infty }\cdot N)\;\phi \;dA+\int_{{ℬ}_{0}}l_{0}^{f}\;dV+\int_{\partial {ℬ}_{0}}l_{0}^{t}\;dV\right\}, & \end{array}$$

whereby we note that, as a consequence of fixing the outer boundary $\partial {ℬ}^{\infty }$, the loading terms on that boundary in (23) and (24) indeed take this form with a slight abuse of notation. Moreover, outside ℬ the energy densities $\Psi_{0}^{B}(C,B)$ and $\Psi_{0}^{B}(C,H)$ reduce to the respective vacuum energy densities discussed in Section 2.4.

### 2.4 Specific relations for non‐magnetic media

In empty space (vacuum) and, to very good approximation, in all (technically) *non‐magnetic* ^#^ media the fields h and b are related through

(25) $$b=\mu_{0}\;h,$$

with $\mu_{0}$ being the vacuum permeability. The corresponding (co‐)energy densities are given as

(26) $$\begin{array}{ll} \Psi_{t}^{\text{vac},b}(b)=\frac{1}{2\mu_{0}}b\cdot b, & \end{array}$$

and

(27) $$\begin{array}{ll} \Psi_{t}^{\text{vac},h}(h)=-\frac{\mu_{0}}{2}h\cdot h, & \end{array}$$

from which (25) can be easily computed via (18)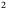 or (21)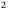, respectively.

An important consideration in this context is that non‐magnetic media do not experience any “magnetic forces.” This means that they will not move or deform under magnetic field. Also, in general they remain non‐magnetic under deformation.

This is reflected by the following observation: consider any of the energy densities (26) or (27) and employ them in the respective magnetomechanical variational principle (23) or (24). For this purpose, the densities are expressed in their Lagrangian form

(28) $$\begin{array}{ll} \Psi_{0}^{\text{vac},B}(C,B)=\frac{1}{2J\mu_{0}}B\cdot (C\cdot B), & \end{array}$$

and

(29) $$\begin{array}{ll} \Psi_{0}^{\text{vac},H}(C,H)=-\frac{J\mu_{0}}{2}H\cdot (C^{-1}\cdot H). & \end{array}$$

Then, *irrespective whether magnetostatic energy or co‐energy is used*, the resulting Cauchy‐type stress field in this particular case is

(30) $$\begin{array}{ll} \sigma^{\text{MW}}:=2\;J^{-1}F\cdot \frac{\partial \Psi^{\text{vac}}}{\partial C}\cdot F^{T}=\frac{1}{\mu_{0}}b⊗b-\frac{1}{2\mu_{0}}\|b\|\;1=h⊗b-\frac{1}{2}(h\cdot b)1=\mu_{0}h⊗h-\frac{\mu_{0}}{2}\|h\|\;1, & \end{array}$$

which is commonly known as the *Maxwell stress* ^‖^.

The key properties of the Maxwell stress are (i) that its Eulerian form only depends on magnetic quantities but no mechanical (kinematic) ones and (ii) that it is divergence‐free for any magnetostatic solution in non‐magnetic media. Combining both reveals that there cannot be any magnetic force densities in such a medium irrespective of the deformation state. An equivalent viewpoint is that Eulerian magnetostatic energy an co‐energy are invariant with respect to deformation in the interior of a non‐magnetic medium.

Remark 1 The simplicity of the vacuum constitutive relation (25), in particular the scalar appearance of $\mu_{0}$, hides the fact that h and b are of different geometric nature. By “pulling‐back” this equation to the Lagrangian configuration, we obtain

(31) $$\begin{array}{ll} B=JF^{-1}\cdot (\mu_{0}F^{-T})\cdot H=(JC^{-1}\mu_{0})\cdot H, & \end{array}$$

which reveals the tensorial object represented by $\mu_{0}$. In terms of differential geometry, h corresponds to a one‐form whereas b corresponds to a two‐form, which roughly means that the former is a “gradient‐type” field and the latter is of “curl‐type.” Both can be expressed as vectors, but they transform differently. A tensorial object that relates fields of different character in the above sense is an instance of a Hodge operator.

### 2.5 Magnetic media and magnetic forces

In accordance with the above “definition” of a non‐magnetic medium we regard any medium for which (25) does not hold in general as *magnetic* or *magnetized*. The mismatch is connected to the magnetization m which appears as

(32) $$\begin{array}{ll} b=\mu_{0}(h+m), & \end{array}$$

such that non‐magnetic media have m≡0.

It is important to note here that the generic constitutive relations (18)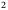 and (21)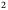 still hold. In particular,^95^

(33) $$\begin{array}{ll} & m=\frac{\partial \Psi_{t}^{\text{vac},b}(b)}{\partial b}-\frac{\partial \Psi_{t}^{b}(F,b)}{\partial b}=\frac{\partial \left(\Psi_{t}^{\text{vac},b}(b)-\Psi_{t}^{b}(F,b)\right)}{\partial b}=-\frac{\partial \Psi_{t}^{\text{mat},b}(F,b)}{\partial b}, \end{array}$$

and

(34) $$\begin{array}{ll} & \mu_{0}m=\frac{\partial \Psi_{t}^{\text{vac},h}(h)}{\partial h}-\frac{\partial \Psi_{t}^{h}(F,h)}{\partial h}=\frac{\partial \left(\Psi_{t}^{\text{vac},h}(h)-\Psi_{t}^{h}(F,h)\right)}{\partial h}=-\frac{\partial \Psi_{t}^{\text{mat},h}(F,h)}{\partial h}, \end{array}$$

where $\Psi_{t}^{b}(F,b)$ and $\Psi_{t}^{h}(F,h)$ are *total* energy densities including the vacuum energy densities whereas $\Psi_{t}^{\text{mat},b}(F,b)$ and $\Psi_{t}^{\text{mat},h}(F,h)$ are contributions from matter.

Concerning stresses and force densities due to the presence of magnetic fields, we consider (Table 1)^41^

(35) $$\begin{array}{ll} & \sigma^{\text{mag},b}=\sigma^{\text{MW}}+\frac{\partial \Psi_{t}^{\text{mat},b}(F,b)}{\partial b}\cdot \frac{\partial J^{-1}F\cdot B}{\partial F}\cdot F^{T}=h⊗b-\frac{\mu_{0}}{2}(\|h\|-\|m\|)1, \end{array}$$

(36) $$\begin{array}{ll} & f^{\;\text{mag},b}=-\text{div}\;\sigma^{\text{mag},b}=m\cdot (\text{grad}\;b), \end{array}$$

and

(37) $$\begin{array}{ll} & \sigma^{\text{mag},h}=\sigma^{\text{MW}}+\frac{\partial \Psi_{t}^{\text{mat},b}(F,h)}{\partial h}\cdot \frac{\partial F^{-T}\cdot H}{\partial F}\cdot F^{T}=h⊗b-\frac{\mu_{0}}{2}\|h\|\;1, \end{array}$$

(38) $$\begin{array}{ll} & f^{\;\text{mag},h}=-\text{div}\;\sigma^{\text{mag},h}=\mu_{0}(\text{grad}\;h)\cdot m, \end{array}$$

where we note that results for the divergence were obtained for curlh=0. The fact that the stress contributions are obviously not divergence‐free can be interpreted as the presence of magnetic volume force densities. The corresponding surface force densities at an interface to a non‐magnetic medium are (Table 1)^41^

(39) $$\begin{array}{ll} \left(\sigma^{\text{MW}}{|}_{\text{out}}-\sigma^{\text{mag},b}{|}_{\text{in}}\right)\cdot n=\frac{-\mu_{0}}{2}\left[{\left(m\cdot n\right)}^{2}+\left(m\cdot m\right)\right]n, & \end{array}$$

and

(40) $$\begin{array}{ll} \left(\sigma^{\text{MW}}{|}_{\text{out}}-\sigma^{\text{mag},h}{|}_{\text{in}}\right)\cdot n=\frac{-\mu_{0}}{2}{\left(m\cdot n\right)}^{2}\;n, & \end{array}$$

where n is the *outward pointing normal vector*.

**TABLE 1:** Material parameters for the solid domains in the bilayer beam example.

Domain	G(kPa)	$G^{\prime }\;(\text{kPa})$	$\rho (\mathrm{kg}\;m^{-3})$	χ(1)	$m^{s}(\mathrm{mA}\;m^{-1})$
${ℬ}^{\text{magn}}$	$2\times 10^{3}$	$100\times 10^{3}$	$2\times 10^{3}$	10	1
${ℬ}^{\text{nonm}}$	1	50	$1\times 10^{3}$	0	–

Remark 2 In magnetically anisotropic media, that is, media where b is not necessarily parallel to h, the magneto‐mechanical Cauchy‐type stresses (35) and (37) are not symmetric but only the *total* Cauchy‐type stress (11) is. This corresponds to the presence of magnetic torques. Examples for such materials are MREs with anisotropic particle distributions^6^, ^96^ and h‐MREs when remanently magnetized. We refer to Mukherjee et al.^77^ for a related discussion of the latter case.

When considering two rigid magnetic bodies that are separated by a non‐magnetic medium, there will be some kind of force transmitted between these two bodies. The transmission indeed happens through the non‐magnetic medium. In fact, the net magnetic force ${ℱ}^{\text{mag}}$ on a magnetized body can be computed by the surface integral of the normal component of the Maxwell stress over any closed surface 𝒮 around the body of interest^35^

(41) $$\begin{array}{ll} {ℱ}^{\text{mag}}=\int_{{𝒮}_{t}}\sigma^{\text{MW}}\cdot n\;da. & \end{array}$$

**FIGURE 3:**
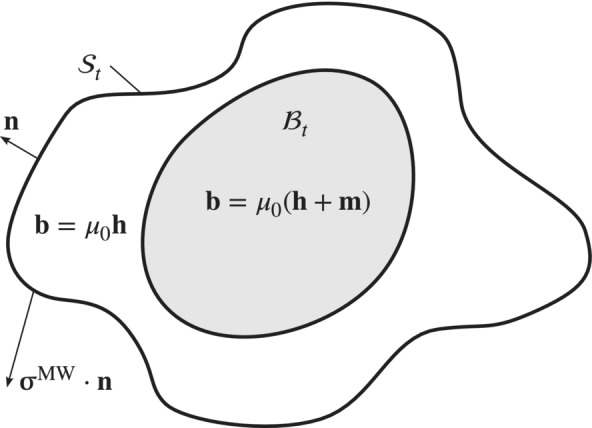
A magnetized body ${ℬ}_{t}$ surrounded by a non‐magnetic medium. The closed surface ${𝒮}_{t}$ lies in the non‐magnetic domain and encloses the magnetic body.

See Figure 3 for an illustration.

Orthogonal to that is the important case of a magnetized body that does not experience a net magnetic force. This can be a body with remanent magnetization that is remote from other magnetic media or a uniformly magnetized body in a uniform external field. Then, even in absence of a net force, there will still be jumps in the normal component of the magneto‐mechanical stress across the boundary of the magnetic body that depend on the magnetization m (see Equations 39 and 40). In other words, the body still experiences tractions that are equilibrated in the sense that the net force vanishes. However, they may still cause deformation. The overall effect of these traction on a body depends on its shape, which is the fundamental mechanism behind the shape‐dependent magneto‐mechanical response of MREs.^51^ A corresponding energetic point of view has been put forward by the same authors.^97^ Such settings are also formidable test cases for the present study because of the strong coupling of boundary deformation and magnetic response even in geometrically “simple” settings.

**FIGURE 4:**
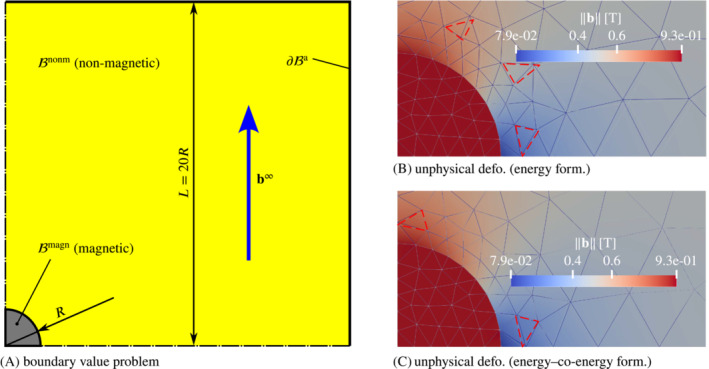
Test problem demonstrating a mild case of spurious deformation in a non‐magnetic deformable elastic domain. Subplot (A) depicts the (quarter) BVP of a rigid magnetic particle ${ℬ}^{\text{magn}}$ embedded in a non‐magnetic elastic medium ${ℬ}^{\text{nonm}}$ exposed to a uniform external magnetic field $b^{\infty }$. Subplot (B) shows the result obtained with the magneto‐mechanical energy formulation (23) with color‐contours indicating to the magnetic field magnitude. The blue mesh corresponds to the deformed configurations, whereas the gray lines indicated the undeformed mesh. The dashed red triangles highlight parts of the *undeformed* configuration which are significantly deformed under applied field. The corresponding plot for the energy‐co‐energy formulation (24) is depicted in subplot (C).

## 3 SPURIOUS MAGNETO‐MECHANICAL COUPLING

While the theory clearly states that non‐magnetic media do not deform in reaction to magnetic fields, one may observe the contrary in numerical simulations. For the purpose of a brief demonstration, we consider the magneto‐mechanical (quarter) BVP of a circular quasi‐rigid magnetic body surrounded by a rather soft non‐magnetic medium such as a very soft elastomer or some extremely soft auxiliary material representing air. The geometrical setting is illustrated by Figure 4A. The BVP is formulated in terms of both coupled variational principles (23) and (24), which are discretized with second‐order isoparametric Lagrange elements for both the deformation (displacements) and the out‐of plane vector potential component or the scalar magnetic potential, respectively, as commonly done in finite magneto‐ and electro‐elasticity.^44^, ^46^, ^49^, ^50^, ^52^, ^84^, ^98^ The resulting fully coupled monolithic nonlinear system obtained is solved by a Newton‐Raphson procedure. Before we continue, we need to clarify some terms. With “fully coupled” we simply refer to schemes which do not neglect any coupling that comes out of the variational principle to be discretized, that is, no physical effects present in the theory are neglected or dropped for computational considerations or convenience. Second, “monolithic” means that one solves the equations representing the fully coupled discretized system for all primary variables at the same time in the whole discretized spatial domain. For example, in the case of (24), one solves for the pair {φ,ϕ} in all magnetic and non‐magnetic media under consideration.

Below we consider both the energy ({φ,A}) and energy–co‐energy formulations ({φ,ϕ}). For both the magnetic and non‐magnetic domain we consider the energy densities

(42) $$\begin{array}{ll} \Psi_{0}^{B}=\frac{G}{2}\left[\text{Tr}\;C-2\log J-3\right]+\frac{G^{\prime }}{2}{\left(J-1\right)}^{2}-\frac{\chi }{2J\mu_{0}(1+\chi )}B\cdot (C\cdot B)+\Psi_{0}^{\text{vac},B}, & \end{array}$$

and

(43) $$\begin{array}{ll} \Psi_{0}^{H}=\frac{G}{2}\left[\text{Tr}\;C-2\log J-3\right]+\frac{G^{\prime }}{2}{\left(J-1\right)}^{2}-\frac{J\mu_{0}\chi }{2}H\cdot (C^{-1}\cdot H)+\Psi_{0}^{\text{vac},H}, & \end{array}$$

where vacuum permeability is $\mu_{0}=0.4\pi \;\mathrm{\mu T}\;mA^{-1}$. For either formulation, the material parameters employed for the practically rigid magnetic domain are $\left\{G,G^{\prime },\chi \}=\{1\times 10^{4}\;\text{kPa},5\times 10^{5}\;\text{kPa},10\right\}$. For the non‐magnetic medium we choose $\left\{G,G^{\prime },\chi \}=\{1\times 10^{-5}\;\text{kPa},5\times 10^{-4}\;\text{kPa},0\right\}$ when using the energy formulation but $\left\{G,G^{\prime },\chi \}=\{1\times 10^{-3}\;\text{kPa},5\times 10^{-2}\;\text{kPa},0\right\}$ in the energy–co‐energy formulation. For the non‐magnetic domain, we for the purpose of demonstration employ different mechanical parameters because the spurious coupling is more pronounced for the energy–co‐energy formulation in this example.

**FIGURE 5:**
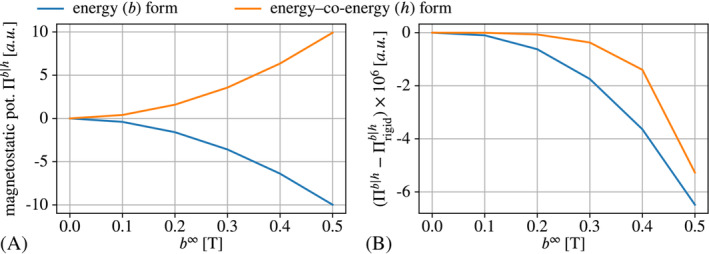
Magnetostatic (co‐)energy $\Pi^{b|h}$ for a rigid magnetic particle embedded in a non‐magnetic solid depending on the applied field magnitude $b^{\infty }$ (A). The respective difference to the (co‐)energy $\Pi_{\text{rigid}}^{b|h}$ obtained for a *rigid* non‐magnetic domain (B).

Figure 4B,C show color‐contours of the magnetic field magnitude in the deformed configuration obtained with the magneto‐mechanical energy formulation (23) and the energy–co‐energy formulation (24), respectively. The blue mesh corresponds to the deformed configuration while the light gray mesh is the undeformed one. Both meshes coincide in the practically rigid magnetic particle. From the physical theory one would not expect any deformation or displacements in the non‐magnetic domain, since the magnetic body is spatially fixed and rigid whereas the deformable material is non‐magnetic and clamped at the outer boundary $\partial {ℬ}^{a}$. However, the dashed‐red triangles marking undeformed triangle cells which undergo significant deformation (dashed red vs. solid blue) show that the opposite is the case. This clearly is against the physical theory and, as we argue below, a universal trait of fully coupled monolithic discretizations. The fundamental problem with such approaches is that the solution fields differ for each discretization, which is an inherent property of any numerical method in general. This means that, the values of the governing energies or potentials evaluated for numerical solution states change with the discretization. The crucial step is now to consider the magnetostatic (co‐)potentials

(44) $$\begin{array}{ll} \Pi^{B}(\varphi ,A) & =\int_{{ℬ}_{0}^{\infty }}\Psi_{0}^{\text{vac},B}(F,B)\;dV-\int_{{ℬ}_{0}^{\text{magn}}}\frac{\chi }{2J\mu_{0}(1+\chi )}B\cdot (C\cdot B)\;dV-\int_{\partial {ℬ}_{0}^{\infty }}(h^{\infty }\times n)\cdot A\;dA\\ & =\int_{{ℬ}_{t}^{\infty }}\Psi_{t}^{\text{vac},b}(b)\;dv+\int_{{ℬ}_{t}^{\text{magn}}}\frac{\chi }{2\mu_{0}(1+\chi )}\|b{\|}^{2}\;dv-\int_{\partial {ℬ}_{t}^{\infty }}(h^{\infty }\times n)\cdot a\;da=\Pi^{b}(a;\varphi ), \end{array}$$

and

(45) $$\begin{array}{ll} \Pi^{H}(\varphi ,\phi ) & =\int_{{ℬ}_{0}^{\infty }}\Psi_{0}^{\text{vac},H}(F,H)\;dV-\int_{{ℬ}_{0}^{\text{magn}}}\frac{J\mu_{0}\chi }{2}H\cdot (C^{-1}\cdot H)-\int_{\partial {ℬ}_{0}^{\infty }}(B^{\infty }\cdot N)\phi \;dA\\ & =\int_{{ℬ}_{t}^{\infty }}\Psi_{t}^{h}(h)\;dv-\int_{{ℬ}_{t}^{\text{magn}}}\frac{\mu_{0}\chi }{2}\|h{\|}^{2}\;dv-\int_{\partial {ℬ}_{t}^{\infty }}(b^{\infty }\cdot n)\phi \;da=\Pi^{h}(\phi ;\varphi ), \end{array}$$

respectively, where φ is considered as a “given” parameter in $\Pi^{b}$ and $\Pi^{h}$. Recall from Sections 2.2 and 2.3 that—for a given φ—the magnetostatic problem is solved for a by minimizing $\Pi^{b}$ and for ϕ by maximizing $\Pi^{h}$, respectively. However, we also solve for φ via the minimization of the total (co‐)energy^**^ $\Pi^{B|H}$. When we were not in possession of numerical but exact magnetostatic solutions, $\Pi^{B|H}$ would be invariant with respect to φ in the nonmagnetic domain (the magnetic domain is considered to be rigid in the present example). But as our numerical solutions are not exact, some deformation has to happen^††^ as we have seen in Figure 4B,C. The energetic considerations are illustrated by Figure 5, where subplot (A) shows the magnetostatic potentials $\Pi^{b|h}$ at a solution state and (B) depicts the difference to the rigid case. The graphs in Figure 5A are practically mirrored (neglecting FE errors) as is expected from the duality of the energy and the co‐energy formulations in the case of linear magnetic materials. In Figure 5B, we observe that both potentials $\Pi^{b}$ and $\Pi^{h}$ are smaller for the deformable non‐magnetic medium than for the rigid case, that is, there was a minimization of the magnetostatic energy through deformation. In the energy formulation this is *formally* favorable as we interpret a smaller computed total energy as being closer to the (true) solution than a higher total energy. But of course, this minimization is only possible because of numerical errors and the resulting deformation is entirely unphysical and undesirable. In the energy–co‐energy case, we do not have any “formally” favorable outcome. In particular, the deformation pattern is to our experience in general quite different from the one obtained for the energy formulation. Moreover, it is typically more pronounced—recall that we had $G=1\times 10^{-3}\;\text{kPa}$ in the energy‐co‐energy formulation but $G=1\times 10^{-5}\;\text{kPa}$ in the energy formulation. In the present example, the energy–co‐energy would crash for applied field magnitudes somewhere between around $b^{\infty }=0.5\;T$ and $b^{\infty }=0.6\;T$. In contrast, the energy formulation runs through until at least $b^{\infty }=1.0\;T$. While the latter appears to be more robust, it might also crash eventually. However, if the soft non‐magnetic medium is indeed a physical material and not some auxiliary model for an air‐like environment, any spurious deformation must be avoided. In the next section we present two approaches that achieve this goal.

Remark 3 We have demonstrated spurious deformations by means of an example in a two‐dimensional setting. The underlying mechanism that is connected to discretization errors is obviously also present in three dimensions. In fact, the three‐dimensional settings one can expect even stronger effects because of the stronger nonlinearity in the magnetostatic solutions. For example, the decay of the scalar magnetic potential in the vicinity of a magnetized sphere is faster and more non‐linear than the decay in the vicinity of an infinitely extended cylinder.

Remark 4 The above considerations have parallels to those behind variational r‐adaptivity for finite elasticity.^99^, ^100^, ^101^ In that context, the “spatial motion” (the “usual”) finite strain elasticity problem on the Lagrangian configuration plays a role comparable to the magnetostatic problem in the Eulerian configuration. The Lagrangian mesh optimization, on the other hand, is governed by the “material motion” problem which is parallel to the “spatial” motion problem—in a sense *Eulerian mesh optimization*—in the present context. We furthermore point out that the “material motion” problem corresponds to configurational mechanics and the respective forces are configurational forces.^102^, ^103^, ^104^ They are expected to vanish everywhere except at material *defects* such that one may attain the viewpoint that the discretization of a body indeed introduces defects.

## 4 TWO NOVEL APPROACHES TO CURE SPURIOUS MAGNETO‐MECHANICAL COUPLING IN NON‐MAGNETIC MEDIA

Without loss of generality, we from now on restrict ourselves to the use of the magnetostatic co‐energy and the variational principle (24). Nevertheless, we emphasize that all derivations and observations presented below have their equivalent counterpart when (23) is employed instead. In particular, we recall that the Maxwell stress has a unique notion in non‐magnetic media. As a result, the methods introduced in this Section are directly applicable to both the energy and energy–co‐energy formulation.

Below we shall distinguish two cases. First, we consider magnetic bodies surrounded by vacuum or air, that is, a non‐magnetic medium of practically negligible stiffness. Then we do not have to care too much about the actual deformation outside the bodies. What matters is that the deformation is acceptable from a numerical perspective. The second case is concerned with magnetic bodies that are embedded in a very soft carrier medium such as an extremely soft elastomer, a gel or some biological tissue. In that case one expects a specific mechanical response of non‐magnetic material such that we are not free to choose the mechanical parameters. Because of this constraint, we regard the latter case as more difficult.

To our best knowledge, up to now, all remedies to spurious magneto‐mechanical coupling in non‐magnetic media are concerned with spurious coupling in vacuum or air‐like domains. Therefore, we start with this important case, where we first briefly review existing approaches and then propose two novel cures to this issue. They will be assessed by a comparison with each other and the staggered scheme of Pelteret et al.^84^ After that demonstration, in Section 4.5 we will turn to the extension of the proposed methods to extremely soft, non‐magnetic solids.

Before jumping into the details, we remark that we only consider the case when spurious deformations are an issue only in the non‐magnetic domains but not in the magnetic ones. In other words, throughout this work we assume magnetic domains to be sufficiently stiff such that spurious deformations are negligible. We anticipate though, that this might not always be the case.

### 4.1 Eliminating spurious coupling in vacuum and air‐like domains

In case of vacuum or air surrounding the body of interest, one might look into schemes which just do not solve the coupled problem in a monolithic but in a staggered way along the lines of Pelteret et al.^84^ In their scheme, one first solves the fully coupled problem in a monolithic way but with deformations blocked in the interior of the non‐magnetic domain. By that one ends up with a thin deformable layer between the boundary of the solid bodies and the first FE nodes in the vacuum domain with vanishing stiffness. As a consequence, this first part of problem is free of any artificial elastic model for the air or vacuum domain. The second step consists of smoothing the displacement field in the vacuum domain by an auxiliary problem. While this adaption does not have any effect on the stiffness experienced by the solid bodies from their “empty” surrounding, there is an effect on the magnetostatic solution due to the change of the Eulerian configuration. And since the problem is physically coupled, the mesh adaption step perturbs the equilibrium computed in the first, coupled step. This can be a disadvantage when very soft bodies or compliant (slender) structures are considered. The situation can be improved by iterating between the coupled and the displacement‐smoothing step.

A second highly successful scheme for the treatment of vacuum or air in this context is the use of non‐local algebraic constraints that bind the displacement in the vacuum domain to the motion of the boundaries of the embedded bodies.^86^ This scheme is in fact monolithic and thus does not suffer from the convergence issues of the staggered approach discussed before. However, the non‐local constraints can be difficult to setup for complex geometries^105^ and furthermore lead to an unfavorable sparsity structure of the system matrix. Both, the staggered and the non‐local constraint approaches—if applicable—lead to quantitatively better results than the probably most straightforward method from an implementation perspective: assigning a small but sufficient artificial stiffness to the vacuum domain.^50^, ^105^ By that one ends up with a perturbed BVP similar to the one employed in Section 3 for the demonstration of spurious magnetic forces. We will refer to this approach as “naive monolithic scheme.” As we have seen in Section 3, spurious coupling may eventually lead to crashes. Thus, for the “naive monolithic scheme” we face the problem that the spurious coupling sets a lower bound on the (auxiliary) stiffness added. Since the accuracy of the “vacuum” approximation depends on the ratio of “solid” to “vacuum” stiffness, the lower bound on the auxiliary stiffness will at some point lead to significant deviations from the unperturbed problem and its solution. The “naive” approach can be significantly improved by adapting the stiffness added to the magnitude and the “profile” of the magnetic fields in the vacuum domain as demonstrated recently.^76^ However, this requires a certain knowledge of the problem or complicated heuristics to automatically assign optimal stiffness parameters. Moreover, such improvements only shift the limit but do not solve the underlying problem.

A somewhat distinct method is that of Liu et al.,^85^ which is essentially based on magnetic forces and tractions derived from numerically computed fields and (localized and free) electric currents. By that, no forces emerge in non‐magnetic domains. In order to adapt the deformation of the vacuum domain to that of the slender structure under consideration they thus may assign a much smaller—indeed negligible—artificial stiffness. However, their specific scheme for the computation of magnetic volume and surface force densities requires to compute derivatives of the magnetization, which are not directly available in standard FE simulations and thus prevents a monolithic approach. Instead, their solution procedure is a staggered one based on the alternate solution of the magnetostatic and the elasticity problem. Hence, one needs a certain number of iterations between the two subproblems in order to solve the coupled problem.

What we propose below has partially great similarities with the method of Liu et al.^85^ on a higher level in the sense that we focus on what is going on at the interface between magnetic and non‐magnetic bodies. However, in contrast to Liu et al.,^85^ our schemes essentially rely on the same building blocks as the naive monolithic scheme. By that the proposed methods can be implemented with minimal adaptions to existing code. In particular, there is no need to compute forces emerging from currents nor anything else that would require a staggered algorithm. Thus, the new schemes can be easily linearized and will be implemented in a monolithic way. When starting from an implementation of the naive monolithic scheme, only minor adaptions of code are required.

### 4.2 The Maxwell traction approach

For the derivation of the first approach we consider the “mechanical” weak form corresponding to the first variation of the governing potentials with respect to deformation restricted to vacuum domains ${ℬ}^{a}$. We emphasize that at this stage, the only (co‐)energy density considered in vacuum is the magnetostatic one. Thus, the only stress present is the vacuum Maxwell stress (30) and the “mechanical” weak forms for the energy and the co‐energy versions coincide

(46) $$\begin{array}{ll} \delta_{\varphi }\int_{{ℬ}_{0}^{a}}\Psi_{0}^{\text{vac},B}(C,B)\;dV=\delta_{\varphi }\int_{{ℬ}_{0}^{a}}\Psi_{0}^{\text{vac},H}(C,H)\;dV=\int_{{ℬ}_{t}^{a}}\sigma^{\text{MW}}:\text{grad}\;\delta \varphi \;dv. & \end{array}$$

As discussed in the context of (30), $\sigma^{\text{MW}}$, the vacuum Maxwell stress is a divergence‐free field such that we may safely^‡‡^ transform the equation above by means of the divergence theorem

(47) $$\begin{array}{ll} \int_{{ℬ}_{t}^{a}}\sigma^{\text{MW}}:\text{grad}\;\delta \varphi \;dv=\int_{\partial {ℬ}_{t}^{a}}\delta \varphi \cdot \left(\sigma^{\text{MW}}\cdot n\right)\;da=\int_{\partial {ℬ}_{0}^{a}}\delta \varphi \cdot \left(P^{\text{MW}}\cdot N\right)\;dA, & \end{array}$$

where $P^{\text{MW}}$ is the first‐Piola‐Kirchhoff‐type instance of the Maxwell stress. The important observation here is that, in the case of the FE discretizations under consideration^§§^, the boundary integrals in (47) do contribute any discrete “interior” forces^¶¶^. As a consequence, no spurious coupling within ${ℬ}^{a}$ can emerge from such boundary integrals whereas the volume integral in (47) indeed suffers from this numerical artifact.^##^ This is somehow parallel to the use of magnetic surface forces by Liu et al.^85^ but approached from a quite different angle. Indeed, the jump of the normal components of the “magnetic” stresses corresponds to the magnetic surface tractions (see (35) and (37)), but here we only compute the respective contribution on the vacuum side.

In order to obtain a non‐singular system matrix in FE simulations we equip the vacuum domain with a very soft elastic material. However, in contrast to the “naive” monolithic scheme, the artificial stiffness does not have to counterbalance the effects of spurious forces and is thus only limited from below by requirements of the linear solver and numerical stability, respectively.

Summarizingly, the above considerations ask us to implement the right‐hand‐side of

(48) $$\begin{array}{ll} \delta_{\varphi }\int_{{ℬ}_{0}^{a}}\Psi_{0}^{\text{vac},H}(C,H)+\Psi^{a}(C)\;dV=\delta_{\varphi }\int_{{ℬ}_{0}^{a}}\Psi^{a}(C)\;dV+\int_{\partial {ℬ}_{0}^{a}}\delta \varphi \cdot \left(P^{\text{MW}}\cdot N\right)\;dA, & \end{array}$$

instead of the left‐hand‐side, where $\Psi^{a}$ is the auxiliary strain energy density. Finite element implementations of the boundary integral in (48) approach are briefly outline below. The actual implementations in Netgen/NGSolve are provided as Supplementary Material.

#### 4.2.1 Direct implementation of the boundary integral

The most obvious finite element implementation of the “Maxwell traction” approach is the implementation of the boundary integral in (48) just as what it is—a boundary integral. The essential additional algorithmic ingredients are

- a routine the gathers all element faces that belong to the boundary of the non‐magnetic domain and of which the parent (volume) element belongs to the vacuum domain and;
- the evaluation of the outward‐pointing unit normal on these faces.

For our implementation we choose Netgen/NGSolve which directly provides the outward‐pointing unit normal vector and a symbolic language for the integration over element boundaries. However, as will be shown in the numerical examples in Section 4.4, the convergence for refined meshes of such an implementation is not satisfactory. We also point out that while the boundary integral can readily be linearized to obtain its contribution to the system matrix, this contribution is non‐symmetric.

#### 4.2.2 Implementation via discrete force omission

Alternative to the boundary integral implementation, one can exploit the fact that $P^{\text{MW}}$ is expected to be divergence free in a non‐magnetic domain, such that all resulting discrete “interior” forces can just be ignored. In contrast, the discrete forces corresponding to boundary deformation degrees of freedom balance discrete forces stemming from applied tractions or the body on the other side of an interface. Hence, instead of actually computing the boundary integral in strict sense, we may simply compute the volume integral in (48). This is what one would also do in the “naive” monolithic approach but ignoring all discrete forces resulting from $\delta \Psi_{0}^{\text{vac},H}=P^{\text{MW}}:\delta \varphi$ that correspond to deformation DoFs in the *interior* of ${ℬ}^{a}$. The actual implementation thus only needs to be able to distinguish between discrete forces in the interior of the vacuum domain and its boundary. Depending on the finite element software, the deletion of forces might be done by simply omitting the respective contribution during assembly. Alternatively, one can assemble the residual vectors resulting from $\Psi^{a}$ and $\Psi_{0}^{\text{vac},H}$ separately in a first step. In a second step, the “interior” forces from the latter residual vector are deleted such that only the boundary forces remain. The final residual vector is the obtained as the sum of the auxiliary residual vector and the fleshed‐out vacuum residual. It is important to note that the omission or deletion of discrete force contribution entails the deletion of the corresponding contributions to the system matrix, rendering the latter non‐symmetric.

Our actual implementation employs the latter method as this can be done without any low‐level changes to the assembly routines of NGSolve.

### 4.3 The traction compensation approach for air‐like media

The second approach proposed also relies on tractions but, besides that, builds upon a converse line of thinking. The basic idea is to assign an auxiliary finite strain energy $\Psi^{a}$ to the vacuum domain and then compensate the effect of the stiffness added by subtracting the corresponding *mechanical* traction from the boundary $\partial {ℬ}^{a}$. This means adding an “integral zero” to the functional under the *condition* that the corresponding stress field is *divergence‐ free*. We start from the weak form (46) and proceed as outlined to obtain

(49) $$\begin{array}{ll} & \int_{{ℬ}_{t}^{a}}\sigma^{\text{MW}}:\text{grad}\;\delta \varphi \;dv=\int_{{ℬ}_{t}^{a}}\sigma^{\text{MW}}:\text{grad}\;\delta \varphi \;dv+\underset{=-\int_{{ℬ}_{t}^{a}}\delta \varphi \cdot \left(\text{div}\;\sigma^{a}\right)\;dv=0}{\underbrace{\int_{{ℬ}_{t}^{a}}\sigma^{a}:\text{grad}\;\delta \varphi \;dv-\int_{\partial {ℬ}_{t}^{a}}\delta \varphi \cdot \left(\sigma^{a}\cdot n\right)\;da}} \end{array}$$

with

(50) $$\begin{array}{ll} & \sigma^{a}=J^{-1}\frac{\partial \Psi^{a}}{\partial F}\cdot F^{T}. \end{array}$$

While the right‐hand‐side of (49) is the basis for our implementations, further manipulations provide additional insight into the method:

(51) $$\begin{array}{ll} & \int_{{ℬ}_{t}^{a}}\sigma^{\text{MW}}:\text{grad}\;\delta \varphi \;dv+\int_{{ℬ}_{t}^{a}}\sigma^{a}:\text{grad}\;\delta \varphi \;dv-\int_{\partial {ℬ}_{t}^{a}}\delta \varphi \cdot \left(\sigma^{a}\cdot n\right)\;da\\ & =\int_{{ℬ}_{t}^{a}}\underset{\sigma^{\text{MW}}+\sigma^{a}}{\underbrace{\sigma }}:\text{grad}\;\delta \varphi \;dv-\int_{\partial {ℬ}_{t}^{a}}\delta \varphi \cdot \left(\sigma^{a}\cdot n\right)\;da\\ & =\int_{{ℬ}_{t}^{a}}-\delta \varphi \cdot \left(\text{div}\;\sigma \right)\;dv+\int_{\partial {ℬ}_{t}^{a}}\delta \varphi \cdot \left(\sigma^{\text{MW}}\cdot n\right)\;da. \end{array}$$

From this equation one can see that, irrespective of the actual value of $\sigma^{a}$, only the Maxwell stress is exerted on embedded bodies (“across” the boundaries of the vacuum domain). Thus, as the actual deformation in the *interior* of the vacuum domain does not affect its neighboring bodies, which allows for great freedom in the choice (form and magnitude) of $\Psi^{a}$ or $\sigma^{a}$, respectively. A second aspect is that one effectively solves for $\text{div}\;\sigma =\text{div}\;(\sigma^{\text{MW}}+\sigma^{a})=0$ which allows for $\text{div}\sigma^{a}\neq 0$ such that the auxiliary stiffness can counterbalance any spurious forces corresponding to $\text{div}\sigma^{\text{MW}}\neq 0$. In that case, the terms added in the derivation do not exactly add up to zero.

The crucial aspect from an implementation point of view is the treatment of the boundary integral in (49). In the remainder of this subsection we present two possible finite element approaches for which the actual implementation in Netgen/NGSolve is provided as Supplementary Material.

#### 4.3.1 Direct computation of the traction compensation integral

Similar to the Maxwell traction approach, we start with the direct implementation of (49). The volume integrals are exactly the same as for the “naive” monolithic implementation. The only addition in this sense is the boundary integral in that equation, of which the implementation is technically parallel to that for the Maxwell tractions. It also has the same drawback of bad convergence under mesh refinement as will be shown in the numerical examples in Section 4.4. Similarly, the resulting system matrix is non‐symmetric.

#### 4.3.2 Implementation via discrete force omission

Reusing the viewpoint of force omission approach for Maxwell tractions, one can implement traction compensation via manipulations of the discrete force vector resulting from standard evaluation of volume integrals and the corresponding system matrix contributions. However, in contrast to the Maxwell tractions, now one leaves the discrete forces corresponding to displacements in the interior as they are but removes the auxiliary discrete forces that correspond to displacement DoFs at the boundary of the vacuum domain. This means the we may think of implementing (49) as

(52) $$\begin{array}{ll} & \int_{{ℬ}_{t}^{a}}\sigma^{\text{MW}}:\text{grad}\;\delta \varphi \;dv=\int_{{ℬ}_{t}^{a}}\sigma^{\text{MW}}:\text{grad}\;\delta \varphi \;dv+\int_{{ℬ}_{t}^{a}}\sigma^{a}:\text{grad}\;\delta \varphi \;dv-\underset{\text{omit “interior” forces}}{\underbrace{\int_{{ℬ}_{t}^{a}}\sigma^{a}:\text{grad}\;\delta \varphi \;dv}}, \end{array}$$

or

(53) $$\begin{array}{ll} & \int_{{ℬ}_{t}^{a}}\sigma^{\text{MW}}:\text{grad}\;\delta \varphi \;dv=\int_{{ℬ}_{t}^{a}}\sigma^{\text{MW}}:\text{grad}\;\delta \varphi \;dv+\underset{\text{omit “boundary” forces}}{\underbrace{\int_{{ℬ}_{t}^{a}}\sigma^{a}:\text{grad}\;\delta \varphi \;dv}}. \end{array}$$

Thus, for any implementation the essential algorithmic ingredient is a mean to distinguish “interior” and “boundary” displacement DoFs. Our own implementation first assembles the volume integrals in (53) separately, removes the boundary forces from the auxiliary residual vector and finally adds both vectors. Accounting for the manipulations of discrete forces in the system matrix leads to a loss of symmetry of the latter, as is the case for all approaches proposed in this contribution.

### 4.4 Assessment of the “Maxwell traction” and the “traction compensation approach for air‐like non‐magnetic domains

We critically assess the proposed schemes by their performance in the boundary value problem presented in Section 3, in particular Figure 4A. The energy densities employed are of the form (43). The material parameters for the magnetic domain are $\left\{G,G^{\prime },\chi \}=\{1\;\text{kPa},50\;\text{kPa},10\right\}$. We consider the non‐magnetic domain to be air‐like, where χ=0 and $G=G^{\prime }=0$. However, depending on the numerical scheme applied, we employ different values for the auxiliary hyperelastic parameters $G^{a}$ and ${G^{\prime }}^{a}$. While the effect of their absolute value is to be studied, we keep the ratio of $G^{a}/{G^{\prime }}^{a}=1/50$ throughout all examples. Concerning the discretization we employ triangular meshes and second‐order finite element spaces for each component of the deformation and for the scalar magnetic potential. This appears to be a common choice in literature such that the results reported in this work are directly relevant to research applications.

From a theoretical perspective, the “traction compensation” approach is exact whereas the “Maxwell traction” approach presented is not. This is because in the latter case the effect of the auxiliary stiffness is very small but nevertheless finite. In contrast, all effects from the auxiliary stiffness and body force contributions are compensated in the former case. However, despite being exact in the above sense, the results obtained with the traction compensation approach still depend on the magnitude of added stiffness and forces. This is particular observable for rather small added stiffness such that the spurious coupling may still affect the deformation of the non‐magnetic domains. Therefore, in what follows, we investigate the effect of the absolute value of the auxiliary shear modules $G^{a}$ and the discrete resolution of the domain, that is, the mesh size.

We start the comparison with individual parameter studies of the “naive” monolithic approach, continue with the “Maxwell traction” and “traction compensation” implementations and finally present a comparative convergence study. All results presented below refer to the vertical displacement $u_{2}$ of the point with initial position A=(0,R), that is, the intersection of the boundary of the magnetic domain with the y‐axis, at an applied magnetic field magnitude $b^{\infty }=0.7\;T$.

**FIGURE 6:**
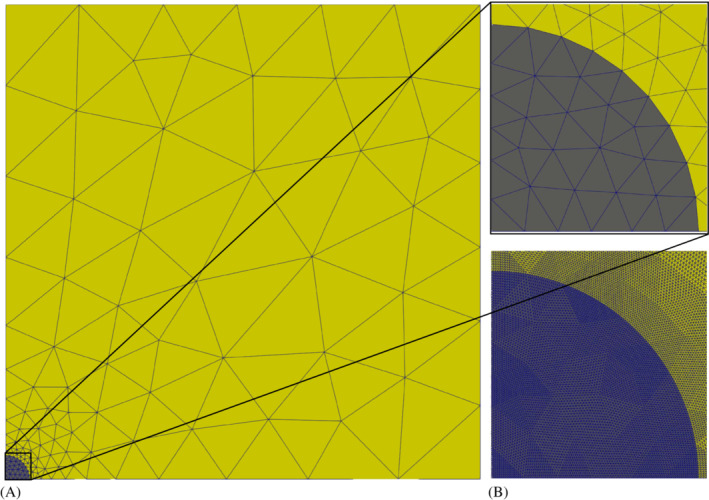
Coarsest and finest mesh of the circular magnetic domain embedded in a non‐magnetic domain. Subplot (A) depicts the “full” (actually quarter domain) mesh. Details of the coarsest and the finest mesh employed are shown in (B). Note that actual geometry representation is of second order, which has only been lost during postprocessing.

The coarsest mesh generated with Netgen is depicted in Figure 6A, a comparison of the coarsest (refinement level zero) and the finest (refinement level four) mesh is provided in Figure 6B. Level zero corresponds to a total of 222 cells and 1473 DoFs. After four refinement steps one has 568 32 cells and 343 203 DoFs.

#### 4.4.1 The “naive” fully‐coupled monolithic auxiliary stiffness approach

**FIGURE 7:**
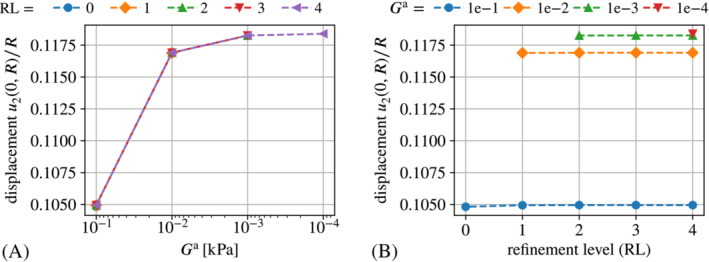
Scaled displacement $u_{2}(0,R)/R$ at $b^{\infty }=0.7\;T$ obtained with the “naive” monolithic auxiliary stiffness approach. The displacement is plotted over (A) the auxiliary free‐space stiffness $G^{a}$ and (B) over the mesh refinement level. The “incomplete” graphs in (A) and (B) reflect the fact that the required auxiliary stiffness decreases with mesh refinement.

For some further insight on the underlying problem of spurious magnetic forces we have a closer look at the capabilities of the “naive” fully‐coupled monolithic auxiliary stiffness approach. Interestingly, as can be seen from Figure 7, the approach works quite well for very fine meshes (refinement level four (RL 4) corresponds to more than $1\times 10^{5}$ DoFs) but not so for coarse meshes. This underlines the fact that spurious magnetic forces result from inaccurate solutions of the magnetostatic part of the problem, which has already been put forward in Section 3. Figure 7 furthermore shows that—if results can be obtained at all—convergence with respect to mesh refinement is only a minor issue compared to the convergence with respect to the auxiliary stiffness parameter $G^{a}$. From a practical point of view, one might say “acceptable” results were obtained already for $G^{a}\leq 1\times 10^{-2}\;\text{kPa}$ and one refinement step. “Accurate” results were only obtained $G^{a}\leq 1\times 10^{-3}\;\text{kPa}$ requiring at least two mesh refinement steps, that is, going from roughly 1000 to 20 000 DoFs. A direct comparison with the methods proposed in this work is presented in Section 4.4.6.

#### 4.4.2 Maxwell traction approach via direct boundary integral computation

**FIGURE 8:**
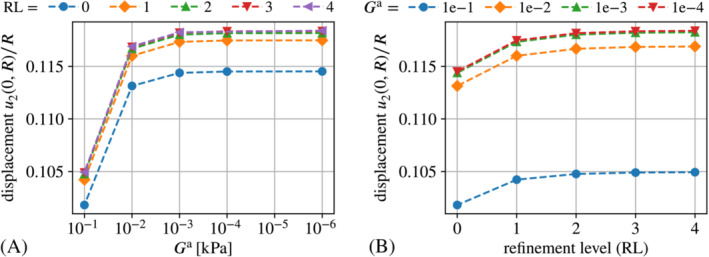
Scaled displacement $u_{2}(0,R)/R$ at $b^{\infty }=0.7\;T$ obtained with the “Maxwell traction” version using boundary integrals. The displacement is plotted over (A) the auxiliary free‐space stiffness $G^{a}$ and (B) over the mesh refinement level. The results for $G^{a}=1\times 10^{-4}$ and $1\times 10^{-6}\;\text{kPa}$ practically coincide such the graph for the latter is omitted in subplot (B).

Figure 8A shows the effect of the auxiliary stiffness parameter $G^{a}$ for different mesh refinement levels whereas subplot (B) shows the effect of the refinement level for given $G^{a}$. From the plots one can see that accurate results are only obtained for $G^{a}\leq 1e-3\;\text{kPa}$ as already observed for the “naive monolithic” approach. Concerning the mesh resolution, three refinement steps (86 355 DoFs) yield a high‐quality solution.

#### 4.4.3 Maxwell traction implementation via discrete force omission

**FIGURE 9:**
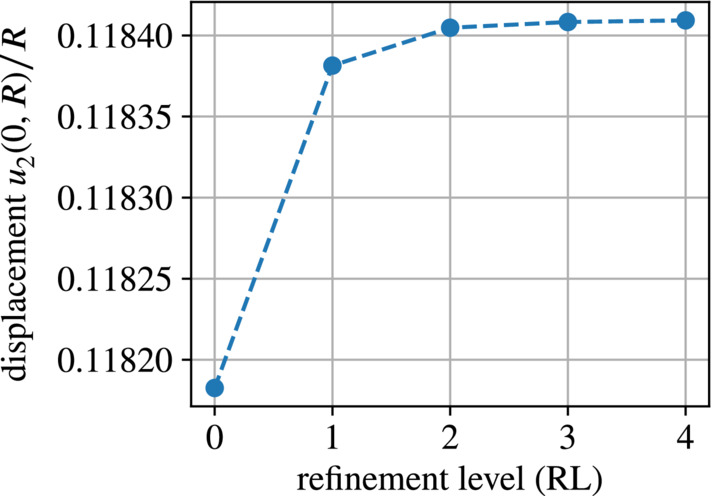
Scaled displacement $u_{2}(0,R)/R$ at $b^{\infty }=0.7\;T$ for an auxiliary stiffness parameter $G^{a}=1\times 10^{-6}\;\text{kPa}$ obtained with the “Maxwell traction” version using volume integrals.

The effect of the auxiliary stiffness parameter $G^{a}$ is essentially the same as in Figure 8A. However, as shown in the mesh convergence plot depicted in Figure 9, the range of displacements is much more narrow than for the boundary integral implementation. This indicates a much higher accuracy of the volume‐integral‐based “discrete force omission” implementation compared to the “direct boundary integral” version. Also, convergence with respect to mesh refinement seems to be attained earlier with the force omission implementation. A study of actual convergence rates is deferred to Section 4.4.6.

#### 4.4.4 Traction compensation implementation via direct boundary integral computation

**FIGURE 10:**
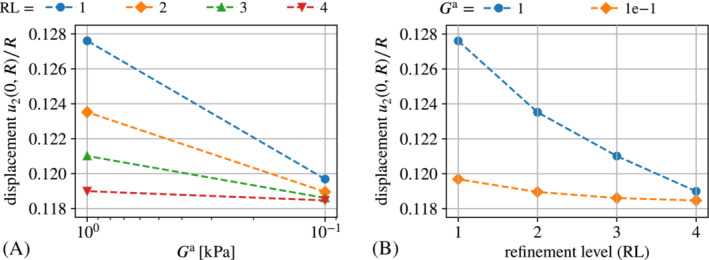
Scaled displacement $u_{2}(0,R)/R$ at $b^{\infty }=0.7\;T$ obtained with the “Traction compensation” approach implemented via direct boundary integral computation. Subplot (A) depicts the displacement plotter over the auxiliary stiffness parameter $G^{a}$ whereas subplot (B) shows the displacement plotter over the mesh refinement level. Note that the parameter $G^{a}$ plays different roles in the “Traction compensation” and “Maxwell traction” such that a comparison of its effect cannot be made directly.

In this case the value of the auxiliary stiffness parameter $G^{a}$ is responsible for preventing excessive deformation due to spurious magnetic forces. Therefore, we employ considerably higher values than for the “Maxwell traction” implementations. Figure 10A

shows a pronounced effect of $G^{a}$ that clearly depends on the mesh resolution. Figure 10B indicated that the value of the auxiliary stiffness $G^{a}$ is of great relevance for coarse meshes but looses its influence for increasingly fine discretizations. For example, with $G^{a}=0.1\;\text{kPa}$ two refinement steps yield approximately equal results as four refinement steps and $G^{a}=1.0\;\text{kPa}$. Thus, lower auxiliary stiffness tends to lead to a significantly higher accuracy which is somewhat unexpected from the theory.

#### 4.4.5 Traction compensation implementation via discrete force omission

**FIGURE 11:**
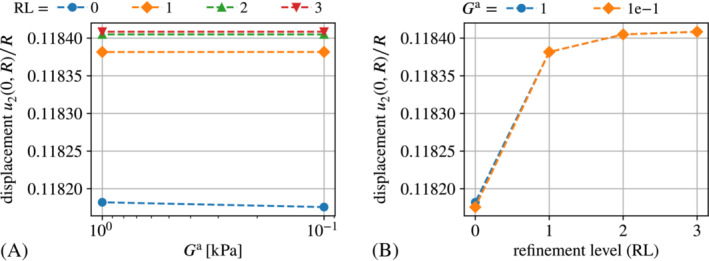
Scaled displacement $u_{2}(0,R)/R$ at $b^{\infty }=0.7\;T$ obtained with the “Traction compensation” approach implemented via volume integrals. Subplot (A) depicts the displacement plotter over the auxiliary stiffness parameter $G^{a}$ whereas subplot (B) shows the displacement plotter over the mesh refinement level.

As can be seen from Figure 11A, the force omission implementation of the traction compensation approach shows almost no sensitivity with respect to the auxiliary stiffness parameter $G^{a}$ as expected theoretically. Only for the coarsest mesh (refinement level zero), one can see a small effect in Figure 11B. This is due to finite deformations caused by spurious magnetic forces in the case of $G^{a}=0.1\;\text{kPa}$. For finer meshes, the spurious forces decline such that the solutions for $G^{a}=0.1\;\text{kPa}$ and $G^{a}=1\;\text{kPa}$ practically coincide.

Also, from the tick‐labels of the y‐axis in both plots one can see that the volume integral implementation overall is much more accurate than the implementation based on direct boundary integral computation.

#### 4.4.6 Comparative convergence study

For the convergence study below, we consider as reference the solution obtained with a staggered scheme along the lines of Pelteret et al.^84^ In order to enhance the accuracy of their scheme, we keep alternating between the coupled and the free‐space adaption sub‐steps until the overall solution is converged. To avoid visual clutter, we only consider one representative set of parameters for each method.

**FIGURE 12:**
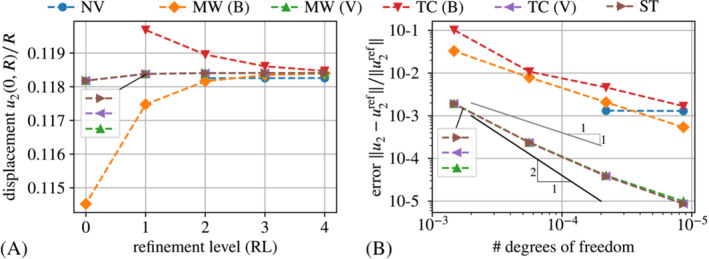
Comparative convergence study in terms of (A) the scaled displacement $u_{2}(0,R)/R$ plotted over the refinement level and (B) the relative displacement error over the number of DoFs in logarithmic axes. The data points in (B) correspond to refinement levels zero to three. The reference solution for (B) is that obtained with a staggered scheme for mesh refinement level four, that is, 343 203 degrees of freedom. The shorthand legend labels expand to: “NV”—“naive” with $G^{a}=1e-3\;\text{kPa}$, “MW (B|V)”—“Maxwell traction” via direct *B*oundary integrals or *V*olume integrals and force omission with $G^{a}=1e-6\;\text{kPa}$, “TC (B|V)”—“Traction compensation” via direct *B*oundary integral or *V*olume integral and force omission with $G^{a}=1\;\text{kPa}$ and “ST”—staggered scheme.

The results for the displacement are shown in Figure 12A.

The plot confirms that the volume integral implementations are much more accurate than their boundary integral counterparts. Indeed, the former practically coincide with the solution obtained with the staggered scheme. The plot in Figure 12B depicts the convergence of the methods under consideration in terms of the deviation from the solution of the staggered scheme at refinement level four (343 203 DoFs) in dependence of the number of DoFs. The solid gray line corresponds to linear convergence and the solid black line to quadratic convergence in mesh resolution. One observes that the volume integral implementations are much more accurate and furthermore converge at super‐linear rate. In contrast, the boundary integral implementations converge at most at a linear rate. Different from all other graphs, the one for the “naive” approach does not exhibit any significant convergence because its solution (or error, respectively) is dominated by the perturbation of the actual problem through the auxiliary stiffness. As a small, but nevertheless noteworthy detail we point out that only for very fine meshes, one can see a small deviation of the “Maxwell traction” implementation via force omission (“MW (B)”) from the staggered and the “traction compensation” via force omission (“TC (B)”). This stems from the auxiliary stiffness $G^{a}=1e-6\;\text{kPa}$ that perturbs the solution in the case of the “Maxwell traction” approach.

Due to the superior performance of the force omission implementations over their boundary integral counterparts, the latter will be discarded from further considerations.

#### 4.4.7 Failure of naive approach

**FIGURE 13:**
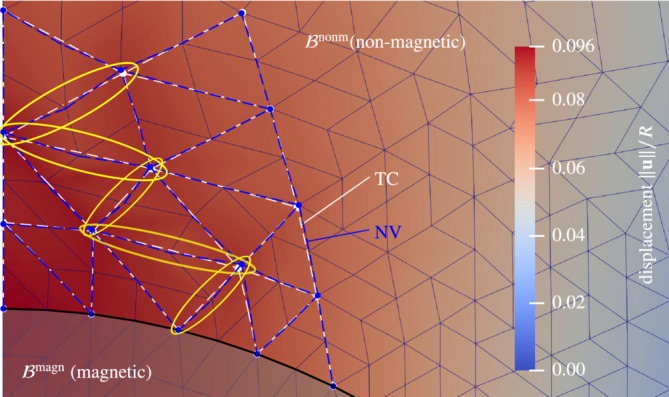
Comparison of the “naive” (NV; $G^{a}=1\times 10^{-3}\;\text{kPa}$) approach with “traction compensation” (TC; $G^{a}=1\;\text{kPa}$) implemented via volume integrals and force omission at applied field $b^{\infty }=0.644625\;T$, where the “naive” scheme looses stability due to spurious coupling. The color contours correspond to the magnitude of displacements obtained with the “naive” approach and so does the thin blue mesh, which is a subdivided and displaced version of the original one. In the region of interest, the dashed white lines refer to the displaced mesh obtained with TC, correspondingly, the dashed blue lines refers to the displaced mesh obtained with NV. Regions where the deformed meshes differ most, are encircled by yellow lines. In order to display the second‐order displacement solutions, the edge midpoints have been displaced accordingly. This does have a visible effect for the blue (NV) mesh but not so for the white (TC) one.

For the example at hand, we found that at the first mesh refinement level (RL1) the “naive” approach with $G^{a}=1\times 10^{-3}\;\text{kPa}$ fails around $b^{\infty }=0.644625\;T$ . Even for tiny load increments the Newton scheme is not at able to reduce the residual down to the prescribed tolerance but stagnates at a certain (small) value, which is typically the case right before loss of stability. For the purpose of demonstration, we assume that the point of stagnation is a perfectly converged solution. In Figure 13 we compare the deformed configurations at $b^{\infty }=0.644625\;T$ for the *almost converged* “naive” approach and the *converged* “traction compensation” (TC) approach via volume integrals and force omission^‖‖^. The white dashed lines correspond to the TC solution, whereas dashed blue lines correspond to the “naive” solution in a region of particular interest. The fact that we regard the non‐converged result of the “naive” approach as a solution is justified by its excellent agreement with the TC solution at the boundary of the magnetic domain (black line). The difference is practically not visible. The deformed meshes have been obtained via subdivision of edges in order to reveal displacements of edge midpoints resulting from the use of second order Lagrange elements. Vertices of the deformed meshes are indicated by white and blue circles. Comparing the deformed meshes, one can observe that there is a certain mismatch between the displacement of vertices. Moreover, while the white circles are connected by practically co‐linear segments, this is in general not the case for the blue mesh. This indicates unphysical excessive local strains obtained that are a manifestation of spurious coupling in the “naive” approach.

### 4.5 Eliminating spurious coupling in non‐magnetic solid domains

In the case of non‐magnetic *solid* domains that are sufficiently soft to suffer from spurious magnetic forces, neither the “staggered”^84^ nor the non‐local constraint^86^ approach can be applied, because both of the methods rely on the absence of physical stiffness. In contrast, both the “Maxwell traction” and “Traction compensation” approach can be extended to non‐magnetic solids.

The adaption of the “Maxwell traction” approach and its implementations to this scenario is straight forward. The auxiliary strain energy density from before is simply substituted by the actual mechanical material model. For the “traction compensation” approach, the modification for solid domains is more delicate and requires a generalization of the fundamental idea: increase mechanical stiffness and compensate this increase by an appropriate increase of loads. Consider the weak balance of momentum for prescribed tractions and body force densities

(54) $$\begin{array}{ll} \int_{{ℬ}_{t}}-\delta \varphi \cdot \left(\text{div}\;\sigma +f^{\;b}\right)\;dv+\int_{\partial {ℬ}_{t}}\delta \varphi \cdot \left(\sigma \cdot n-t\right)\;da=0, & \end{array}$$

where the (total) Cauchy stress σ consists of the Maxwell stress, the mechanical stress resulting from the actual mechanical material model. In order to add a *mechanical* zero holding down spurious magneto‐mechanical interactions, we need to be more careful than before in order to not change the solution of the original problem.

First recall that, within a non‐magnetic material, the presence of magnetic field does not affect the mechanics. Thus, we may factor out anything related to the Maxwell stress, that is,

(55) $$\begin{array}{ll} & \int_{{ℬ}_{t}}-\delta \varphi \cdot \left(\text{div}\;\sigma^{\text{mech}}+f^{\;b}\right)\;dv+\int_{\partial {ℬ}_{t}}\delta \varphi \cdot \left(\sigma^{\text{mech}}\cdot n-t\right)\;da=0, \end{array}$$

and

(56) $$\begin{array}{ll} & \int_{{ℬ}_{t}}-\delta \varphi \cdot \left(\text{div}\;\sigma^{\text{MW}}\right)\;dv+\int_{\partial {ℬ}_{t}}\delta \varphi \cdot \left(\sigma^{\text{MW}}\cdot n\right)\;da=0, \end{array}$$

whereby (56) is automatically fulfilled for any solution of the magnetostatic problem, since the medium under consideration does not experience any magnetic forces. Next, as the purely mechanical part (55) can be treated separately, we may scale it, that is, multiply by some constant factor (1+c), without affecting the solution to this equation. For the purpose of a more compact notation we denote any quantity multiplied by the compensation factor c with a bar, that is, ${\bar{\sigma }}^{\text{mech}}=c\sigma^{\text{mech}}$. Then,

(57) $$\begin{array}{ll} & \int_{{ℬ}_{t}}-\delta \varphi \cdot \left[\text{div}\;\left(\sigma^{\text{mech}}+{\bar{\sigma }}^{\text{mech}}\right)+\left(f^{\;b}+\bar{f^{\;b}}\right)\right]\;dv\\ & +\int_{\partial {ℬ}_{t}}\delta \varphi \cdot \left[\left(\sigma^{\text{mech}}+{\bar{\sigma }}^{\text{mech}}\right)\cdot n-\left(t+\bar{t}\right)\right]\;da=0, \end{array}$$

and any (deformation or displacement) solution to (55) is a solution to (57) and *vice versa*. Doing the usual integration by parts followed by application of the divergence theorem yields

(58) $$\begin{array}{ll} & \underset{=0}{\underbrace{\int_{{ℬ}_{t}}\sigma^{\text{mech}}:\text{grad}\;\delta \varphi +f^{\;b}\cdot \delta \varphi \;dv-\int_{\partial {ℬ}_{t}}\delta \varphi \cdot t\;da}}+\underset{=0}{\underbrace{\int_{{ℬ}_{t}}{\bar{\sigma }}^{\text{mech}}:\text{grad}\;\delta \varphi +\bar{f^{\;b}}\cdot \delta \varphi \;dv-\int_{\partial {ℬ}_{t}}\delta \varphi \cdot \bar{t}\;da}}. \end{array}$$

The first set of terms are contained in the original BVP whereas the second set of terms shall be employed to suppress spurious interactions. In order to proceed towards an implementation that is again based on omission of discrete forces obtained by volume integrals, we have a close look at the second “zero” in (58). The volume integrals can be implemented as they are, but instead of equating them with the boundary integral exploiting $\bar{\sigma }\cdot n=\bar{t}$ we again just omit the resulting discrete forces corresponding to boundary (interface) deformation, that is, the additional terms to be considered are

(59) $$\begin{array}{ll} 0 & =\int_{{ℬ}_{t}}{\bar{\sigma }}^{\text{mech}}:\text{grad}\;\delta \varphi +\bar{f^{\;b}}\cdot \delta \varphi \;dv-\underset{\text{omit discrete “interior” forces}}{\underbrace{\int_{{ℬ}_{t}}{\bar{\sigma }}^{\text{mech}}:\text{grad}\;\delta \varphi +\bar{f^{\;b}}\cdot \delta \varphi \;dv}}, \end{array}$$

or

(60) $$\begin{array}{ll} 0 & =\underset{\text{omit discrete boundary forces}}{\underbrace{\int_{{ℬ}_{t}}{\bar{\sigma }}^{\text{mech}}:\text{grad}\;\delta \varphi +\bar{f^{\;b}}\cdot \delta \varphi \;dv}}. \end{array}$$

In contrast to the traction compensation via discrete force omission for air‐like media, here we employ a multiple of the mechanical strain energy density and a multiple of the body forces which must be scaled by the same factor. Applied tractions, however, do not need any special treatment.

### 4.6 Assessment of the “Maxwell traction” and the “traction compensation approach for non‐magnetic solid domains

In this section, we build upon the trust in the force omission implementations of the “Maxwell traction” and the “traction compensation” approach gained in Section 4.4. The direct boundary integral implementations will not be considered due to their inferior accuracy.

Now, in the case of non‐magnetic solids, we are not any longer in possession of “reference” solutions obtained with the staggered scheme. Instead, we compare the solutions of the proposed methods to results obtained with the “naive” monolithic scheme, which admits direct extension to non‐magnetic solids and is quite accurate and robust as long as the non‐magnetic solid is sufficiently stiff. In fact, what we need to demonstrate in this section is not the accuracy of the new methods for very soft non‐magnetic media as this has already been covered in the previous section. Instead, we want to demonstrate the correctness of the extension to actual solids. Therefore, we may choose material parameters such that a comparison with the “naive” scheme is indeed reasonable.

#### 4.6.1 A magnetic disk in a non‐magnetic carrier under gravitation‐type and magnetic loading

**FIGURE 14:**
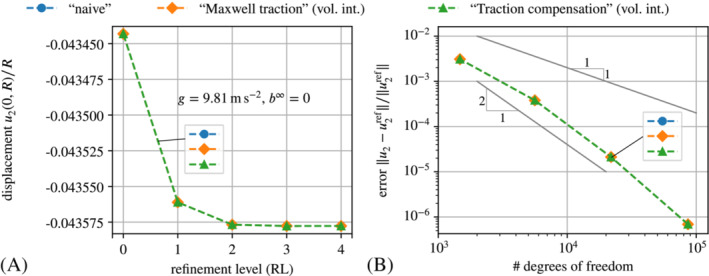
Comparative convergence study for gravitational load ($g=9.81\;m\;s^{-1}$, $b^{\infty }=0$) in terms of (A) the scaled displacement $u_{2}(0,R)/R$ plotted over the refinement level and (B) the relative displacement error over the number of DoFs in logarithmic axes. The data points in (B) correspond to refinement levels zero to three. The reference solution for (B) is that obtained with the “traction compensation” scheme at mesh refinement level four, that is, 343 203 DoFs. Please note that the graph for the “naive” approach is practically hidden behind the other two, which indicates the excellent agreement that is desired in this benchmark.

**FIGURE 15:**
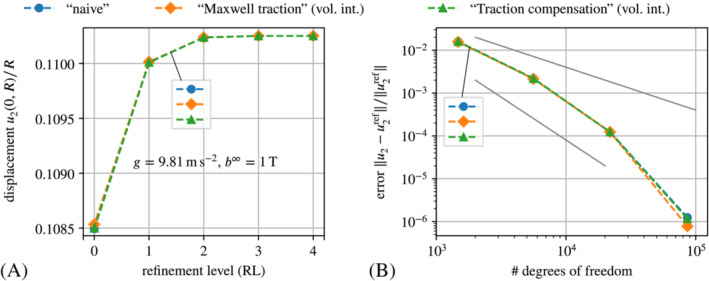
Comparative convergence study for combined gravitational and magnetic load ($g=9.81\;m\;s^{-1}$, $b^{\infty }=1\;T$) in terms of (A) the scaled displacement $u_{2}(0,R)/R$ plotted over the refinement level and (B) the relative displacement error over the number of DoFs in logarithmic axes. The data points in (B) correspond to refinement levels zero to three. The reference solution for (B) is that obtained with the “traction compensation” scheme at mesh refinement level four, that is, 343 203 DoFs. Please note that the graph for the “naive” approach is practically hidden behind the other two, which indicates the excellent agreement that is desired in this benchmark.

**FIGURE 16:**
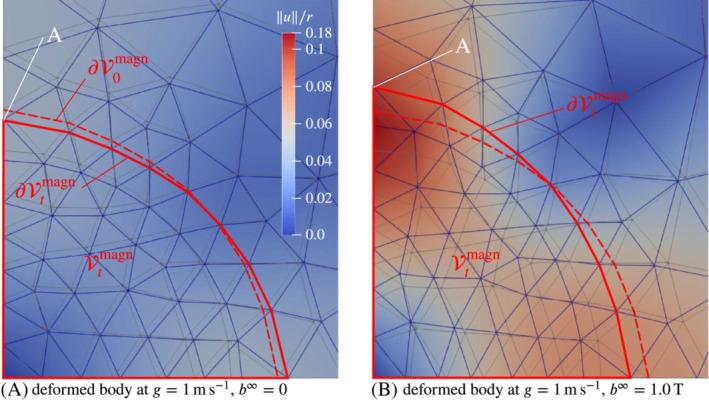
Deformed configurations under (A) purely gravitational load and (B) combined gravitational and magnetic loads. The solid red line indicates the deformed magnetic domain ${ℬ}_{t}^{\text{magn}}$ whereas the dashed red line indicates the boundary of the undeformed magnetic domain $\partial {ℬ}_{0}^{\text{magn}}$. The contours refer to the displacement magnitude, whereby the colors are adjusted to displacement range in (B). Please note that while the mesh nodes are connected by straight lines in the figure, simulations actually have been carried out with second‐order geometry descriptions.

Here we in essence reuse the BVP from Sections 3 and 4.4 but with a specific energy density assigned to the non‐magnetic domain. For simplicity, we again choose an energy density of the form (43) with parameters $\left\{G,G^{\prime },\chi \}=\{1\;\text{kPa},50\;\text{kPa},10\right\}$ for the magnetic domain and $\left\{G,G^{\prime },\chi \}=\{0.5\;\text{kPa},25\;\text{kPa},0\right\}$ for the non‐magnetic domain. In addition, we assign mass densities^***^ to both the magnetic and the non‐magnetic solid leading to gravitation‐like forces. To be specific, the mass density in the magnetic domain is $\rho_{0}^{\text{magn}}=1000\;\mathrm{kg}\;m^{-3}$ whereas in the non‐magnetic domain we employ $\rho_{0}^{\text{nonm}}=100\;\mathrm{kg}\;m^{-3}$. Gravity points in negative vertical (y) direction such that the resulting body forces are given as

(61)

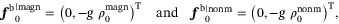


where g denotes the gravitational loading parameter.^†††^ In Figure 14A we show the scaled vertical displacement $u_{2}(0,R)/R$ at $g=1\;m\;s^{-1}$ and $b^{\infty }=0$, that is, purely gravitational loading. The corresponding mesh convergence plot is shown in Figure 14B. In both subplots one can observe the perfect agreement of all three methods. The picture is similar in Figure 15, where the scaled vertical displacement $u_{2}(0,R)/R$ is depicted at the magneto‐mechanical loading state $g=1\;m\;s^{-1}$ and $b^{\infty }=1\;T$. The deformed magnetic bodies at purely gravitational and combined loading are depicted in subplots (A) and (B), respectively, of Figure 16. There one can see that displacement of point “A” is downwards under gravitational load (subplot A) and upwards under combined loading (subplot B).

**FIGURE 17:**
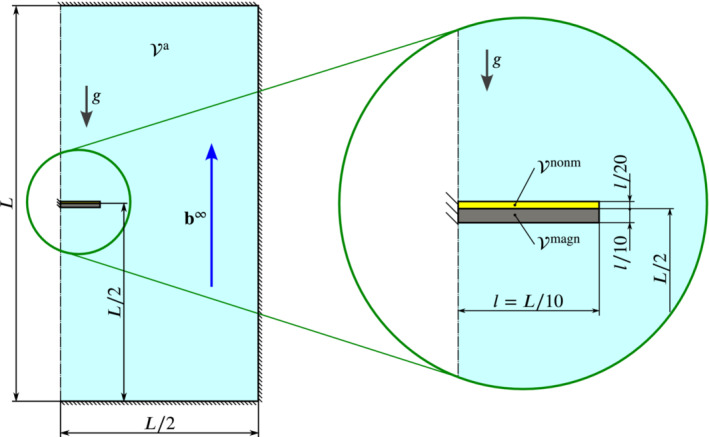
Boundary value problem of a beam that consists of a magnetic ${ℬ}^{\text{magn}}$ and a non‐magnetic layer ${ℬ}^{\text{nonm}}$. It is clamped at its vertical symmetry axis and surrounded by “empty” space ${ℬ}^{a}$. The bilayer beam is exposed to gravity g and a uniform external magnetic field $b^{\infty }$.

**FIGURE 18:**
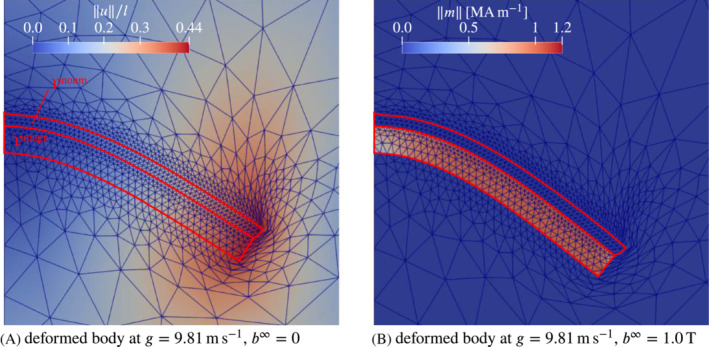
Results for a bilayer beam with a stiffer magnetic layer and a soft non‐magnetic layer exposed to gravity and magnetic field.

#### 4.6.2 A bilayer beam under gravity air magnetic field embedded in an air‐like domain

As final example that demonstrates the capabilities of the proposed schemes we consider a beam consisting of a magnetic and non‐magnetic layer that is embedded in an air‐like domain as depicted in Figure 17. It depicts a bilayer beam consisting of a magnetic ${ℬ}^{\text{magn}}$ and a non‐magnetic layer ${ℬ}^{\text{nonm}}$ that is clamped at its vertical symmetry axis and surrounded by “empty” space ${ℬ}^{a}$. This setting is inspired by prior examples of MRE beams^71^, ^76^, ^77^, ^107^ and the prospective application of magnetoactive materials as mechanically active substrate for biological experiments.^16^ In the simulations, the beam is first exposed to gravity $g=9.81\;m\;s^{-1}$ and in a second step to a combined loading through gravity and a uniform external magnetic field $b^{\infty }=1\;T$. The generic energy density function employed is given as

(62) $$\begin{array}{ll} \Psi_{0}^{H} & =\frac{G}{2}\left[\text{Tr}\;C-2\log J-3\right]+\frac{G^{\prime }}{2}{\left(J-1\right)}^{2}\\ & \;-\frac{J\mu_{0}{\left(m^{s}\right)}^{2}}{\chi }\log\left[\cosh\left(\frac{\chi \sqrt{H\cdot (C^{-1}\cdot H)}}{\mu_{0}m^{s}}\right)\right]+\Psi_{0}^{\text{vac},H}. \end{array}$$

It has the same purely mechanical neo‐Hookean type contribution as in previous examples. The magnetic part, however, now models saturation $\|m\|\to m^{s}$ for ‖h‖→∞.

The material parameters for this example are collected in Table 1. They have been chosen with great care to render a physically reasonable example that challenges the numerical methods under evaluation. Furthermore, when the “Maxwell traction” approach is applied, the air‐like domain ${ℬ}^{a}$ is equipped with $G^{a}=1e-6\;\text{kPa}$. In the case of “traction compensation,” both the non‐magnetic and air‐like domains have $G^{a}=1e2\;\text{kPa}$, which amounts to a compensation factor of c=100 in the solid domain ${ℬ}^{\text{nonm}}$. This means that the auxiliary stiffness added to the solid is much higher that the actual stiffness. This deliberate choice helps us to confirm that all effects from the additional stiffness are compensated properly.

In contrast to all foregoing examples, the geometry of the bilayer beam has sharp corners. This complicates convergence studies because the “naive” scheme has severe problems in such cases. The reason are magnetic field concentrations near the corners leading to pronounced spurious magnetic forces that cannot be simply alleviated with mesh refinement.^83^ ^(Chapter 9, Section 3.1.3)^ Therefore, we omit a rigorous convergence study and instead show contours of solution states in Figure 18. In subplot (A) we observe a gravity‐driven bending‐dominated deformation of the magnetic layer ${ℬ}^{\text{magn}}$ and a slightly more general deformation pattern of the non‐magnetic layer ${ℬ}^{\text{nonm}}$. The color contours in (A) correspond to the deformation magnitude. Subplot (B) shows the deformed configurations under combined loading. The deformation modes are very similar to those under purely gravitational loading, the displacements magnitudes are further increased through the applied magnetic field. The color contours in (B) correspond to the magnetization magnitude. They first of all confirm that only the magnetic layer exhibits magnetization as expected and, second, that the magnetization distribution is far from being perfectly uniform or trivial. This underlines the importance of full‐field simulations of such BVPs. We highlight that in both subplots of Figure 18 one can observe severe deformations of the “empty” domain surrounding the beam in the vicinity of the tip of the beam. While this was not of a great concerns here, in practice one could “smoothen” the deformation field by carefully increasing the auxiliary stiffness in such regions relative to that in other areas of the “empty” domain. Please also note that the post‐processed deformation is only of first order but the actual deformation is the simulation is of second order, such that severely distorted elements in the vicinity of the beam tip are not displayed exactly how they actually appear in the simulations. In any case, the solutions obtained for the Maxwell traction and the traction compensation scheme coincide almost perfectly. In this context we highlight that we did not need to fine‐tune the auxiliary stiffness parameters to achieve excellent agreement between both approaches. This underlines not only their computational robustness but also their robustness with respect to the methods parameters. In general, we for the Maxwell traction scheme recommend to choose the auxiliary stiffness parameter in a range of $10^{-6}$ of the softest solid parameter. For the “Traction compensation” we recommend stiffness parameters roughly in the range of those employed for the magnetic solids.

## 5 CONCLUSION

This work is centered around the issue of spurious magneto‐mechanical interactions that is pervasive in “naive” fully‐coupled numerical simulations of deformable magnetic bodies such as MREs. The key contributions are, first, a thorough characterization of the underlying issue. Second, we present two novel approaches that effectively eliminate or suppress spurious coupling in both vacuum‐ or air‐like media and non‐magnetic solids. The first scheme relies on the so‐called “Maxwell tractions” that removes all unwanted magneto‐mechanical interaction from the interior of the non‐magnetic domain. For definiteness of the solution in “empty” (air‐like or vacuum domains) the method, only needs a negligibly small auxiliary stiffness even in comparison with very soft solids. The second scheme is somewhat dual to the first in that it employs a sufficiently large auxiliary mechanical stiffness to suppress unphysical magneto‐mechanically driven deformation in non‐magnetic domains. The additional stiffness is balanced by additional body force contributions and a removal or compensation of the resulting tractions on the interface to neighboring bodies. The common advantages of our proposed methods over existing successful schemes, in particular in comparison with the staggered schemes^84^, ^85^ and monolithic schemes based on non‐local constraints^76^, ^86^ are twofold. The first is the ease of implementation atop of “naive” monolithic FE simulations because, as has been shown, the successful volume‐integral‐based versions rely on only slight modifications of the weak form. There is no need for modifications to the overall solution procedure, nor cumbersome preprocessing and modifications to the sparsity structure of the linearized system matrix. Second, as demonstrated successfully, both of our approaches are not only applicable for air‐like environments but also for actual, very soft non‐magnetic *solids*. Another advantage over the staggered scheme is that both of the proposed approaches directly allow for the consistent linearization of the fully coupled system, which positively affects the convergence of nonlinear solvers. A mild disadvantage is that the resulting linear systems are non‐symmetric, which increases the effort required for linear solves. To our experience, this is usually outweighed by the improved convergence and robustness of the methods.

We have assessed implementations of both proposed methods regarding their accuracy and their effectiveness regarding the elimination of spurious coupling in comparison with existing approaches. In particular, we have shown the convergence of the “Maxwell traction” approach for decreasing auxiliary stiffness, while the “traction compensation” method converges for increasing auxiliary stiffness. In this context it is important to note that while there remains a parameter to be set, namely the auxiliary stiffness in one form or another, there is typically no need for tuning this parameter with great care. For the “Maxwell traction” one may go right in the range of $10^{-6}$ of the softest solid parameter, for the “traction compensation” one may simply employ some stiffness in the range of the magnetic solids under consideration. The critical comparison with existing schemes has demonstrated the competitiveness of the force‐omission‐based variants of the approaches proposed, with small advantages for the “traction compensation” approach. Thus, in view of the ease of computer implementation and simple choice of parameters both of the proposed methods can hope for wide adoption in future numerical investigations on MREs and related materials or problems, for example, in electromechanics.

## Supporting information


**Appendix S1** Supporting Information

## Data Availability

The data that support the findings of this study, that is, meshes and simulation codes, are openly available in zenodo at https://zenodo.org/record/7533713.

## ENDNOTES

* Note that the fundamental equations of finite electro‐elasticity are formally very similar to those for finite magneto‐elasticity. As a consequence, there was and is a fruitful exchange of computational methods between both fields.

† With *soft* we *roughly* refer to shear moduli in the order of 10kPa or lower, that is, Young's moduli well below 100kPa.

‡ An alternative to truncation of the computational domain is to couple finite element simulations of the solid bodies with boundary elements employed for the solution of the magnetic field equations in the “empty” domain.^90^, ^91^

§ In the present context, one may regard the *initial configuration*, that is, undeformed bodies and media as at the beginning of the deforming process, as reference.

¶ “Fully determined” in the sense of *uniqueness* of $\hat{a}$ in the context of $\hat{b}=\text{curl}\;\hat{a}$.

# Diamagnetism is commonly ignored in the wider context of this contribution.

‖ We will employ “Maxwell stress” only for the particular forms below in vacuum. There is no unique notion of a Maxwell stress in matter such that we prefer avoid the resulting ambiguities.

** We use the short $\Pi^{B|H}$ when we refer to both $\Pi^{B}$ and/or $\Pi^{B}$.

†† The deformation magnitude, of course, depends on the stiffness of the non‐magnetic medium.

‡‡ “Safe” in the sense that the concerns raised by Reich et al.,^106^ who warn from confusing long and short range forces, do not apply.

§§ In this work we are concerned with Lagrange‐type or equivalent elements for the deformation map or displacements, respectively.

¶¶ “Interior” in the sense that the correspond to degrees of freedom (DoFs) that control the deformation in the *interior* of the nonmagnetic domain.

## There is still some spurious coupling within the interface or boundary $\partial {ℬ}^{a}$, but it is assumed that the medium outside is stiff enough such that spurious deformation is suppressed. If this is not the case, the only remedy is sufficiently accurate magnetostatic solution.

‖‖ Note that the TC approach can go well beyond $b^{\infty }=0.644625\;T$. This underlines the fact that the issue faced by the “naive” approach is an unphysical feature of the method.

*** Under assumption of unit length “thickness.”

††† The example serves for the evaluation of the capabilities of the methods under consideration. Thus, we allow ourselves to deviate from actual physical parameters.
